# Organizational principles of amygdalar input-output neuronal circuits

**DOI:** 10.1038/s41380-021-01262-3

**Published:** 2021-08-16

**Authors:** Limeng Huang, Yiwen Chen, Sen Jin, Li Lin, Shumin Duan, Ke Si, Wei Gong, J. Julius Zhu

**Affiliations:** 1 Department of Neurobiology and Department of Neurology of the Second Affiliated Hospital, Zhejiang University School of Medicine, Hangzhou, China; 2 State Key Lab of Modern Optical Instrumentation, College of Optical Science and Engineering, International Research Center for Advanced Photonics, Zhejiang University, Zhejiang, China; 3 Liangzhu Laboratory, Zhejiang University Medical Center, Hangzhou, China; 4 MOE Frontier Science Center for Brain Science & Brain-Machine Integration, NHC and CAMS Key Laboratory of Medical Neurobiology, School of Brain Science and Brain Medicine, Zhejiang University, Hangzhou, China; 5 Department of Pharmacology, University of Virginia School of Medicine, Charlottesville, VA USA; 6 Shenzhen Institutes of Advanced Technology, Chinese Academy of Sciences, Shenzhen, China; 7 School of Pharmaceutical Sciences, Wenzhou Medical University, Wenzhou, China; 8 Research Units for Emotion and Emotion Disorders, Chinese Academy of Medical Sciences, Hangzhou, China

**Keywords:** Psychiatric disorders, Neuroscience

## Abstract

The amygdala, one of the most studied brain structures, integrates brain-wide heterogeneous inputs and governs multidimensional outputs to control diverse behaviors central to survival, yet how amygdalar input-output neuronal circuits are organized remains unclear. Using a simplified cell-type- and projection-specific retrograde transsynaptic tracing technique, we scrutinized brain-wide afferent inputs of four major output neuronal groups in the amygdalar basolateral complex (BLA) that project to the bed nucleus of the stria terminals (BNST), ventral hippocampus (vHPC), medial prefrontal cortex (mPFC) and nucleus accumbens (NAc), respectively. Brain-wide input-output quantitative analysis unveils that BLA efferent neurons receive a diverse array of afferents with varied input weights and predominant contextual representation. Notably, the afferents received by BNST-, vHPC-, mPFC- and NAc-projecting BLA neurons exhibit virtually identical origins and input weights. These results indicate that the organization of amygdalar BLA input-output neuronal circuits follows the input-dependent and output-independent principles, ideal for integrating brain-wide diverse afferent stimuli to control parallel efferent actions. The data provide the objective basis for improving the virtual reality exposure therapy for anxiety disorders and validate the simplified cell-type- and projection-specific retrograde transsynaptic tracing method.

## Introduction

The amygdala, an evolutionarily conserved brain structure, has been intensively examined due to its involvement in a large set of survival behaviors and psychiatric conditions [1–3]. A collective work over the last 70 years has mapped the extensive afferent and efferent connections of the amygdala [4–6]. These data indicate that the amygdala integrates brain-wide diverse afferent stimuli, including sensory, integrative, contextual, neuromodulatory and other miscellaneous inputs. After computation processing, the amygdala emits efferent signals to multiple brain areas, such as the bed nucleus of the stria terminals (BNST), ventral hippocampus (vHPC), medial prefrontal cortex (mPFC) and nucleus accumbens (NAc), to direct multidimensional processes (i.e., initiation, acquisition, evaluation and decision-making) of survival behaviors. However, due to the lack of quantitative and correlative analysis of the brain-wide amygdalar afferent and efferent connections [1], how amygdalar input-output neuronal circuits are organized to transform diverse afferent stimuli into multiple efferent signals to govern survival behaviors remains unclear.

Using a simplified cell-type- and projection-specific retrograde transsynaptic tracing technique, we systematically and quantitatively analyzed afferent and efferent connections of BNST-, vHPC-, mPFC- and NAc-projecting neurons in the primary amygdalar nucleus group, the basolateral complex (BLA). Our analysis reveals that all projecting BLA neurons receive a heterogeneous array of brain-wide afferents with varied input weights and predominant representation of contextual information. Remarkably, BNST-, vHPC-, mPFC- and NAc-projecting BLA neurons receive the brain-wide afferents with virtually identical origins and input weights. The amygdalar BLA afferent and efferent patterns immediately suggest an input-dependent and output-independent anatomical organizational design, which seems to be ideal for integrating brain-wide diverse afferent stimuli to control parallel efferent behavioral actions.

## Materials and methods

### Animals

Postnatal 60 days or older (>P60) male and female wild-type C57BL/6 mice, as well as Vglut2-Cre and Thy1-Cre mice (Jackson Lab, Bar Harbor, MA, stock #016963 and #006143) bred congenically on a C57BL/6 background (Jackson Laboratory, Bar Harbor, ME, USA), were used in this study. The Vglut2-Cre and Thy1-Cre mice were heterozygous for Cre recombinase under control by the *Vglut2* and *Thy1* gene. Genotyping was performed with standard PCR of tail-derived genomic DNAs, with Cre primers 5′-GCG GTC TGG CAG TAA AAA CTA TC-3′ and 5′-GTG AAA CAG CAT TGC TGT CAC TT-3′, and internal positive control primers 5′-CTA GGC CAC AGA ATT GAA AGA TCT-3′ and 5′-GTA GGT GGA AAT TCT AGC ATC ATC C-3′. Mice with specific DNA bands shown at both the Cre and internal positive control positions were used in the experiments. All animals were maintained in the animal facility at the Zhejiang University and family or pair housed in the temperature- (23 ± 1 °C) and humidity- (55 ± 5 %) controlled animal room with 12-h/12-h light/dark cycle. All procedures followed the guidelines for the Care and Use of Laboratory Animals of Zhejiang University approved by the Committee of Laboratory Animal Center of Zhejiang University.

### Viral expression

Viral expression was made similarly as described in our previous reports [7–9]. In brief, animals were anesthetized with sodium pentobarbital (80 mg/kg), and head-fixed in a stereotaxic frame (RWD Life Science, Shenzhen, China). A craniotomy was created above the injection sites with a 0.5 mm diameter drill bit (RWD Life Science, Shenzhen, China). Viral solutions were delivered into various brain areas according to their stereotaxic coordinates with a pulled glass micropipette and pressure injection was made with a Legato 130 syringe pump (KD Scientific Inc., MA, USA) at a rate ~60 nl/min. The injecting micropipette was typically kept in the brain for about 10 min after injection to ensure diffusion of viruses.

For anterograde axon tracing, 80 nl AAV-EF1α-DIO-EYFP-WPRE-pA viral solution with the titer of 1 × 10^12^ vg/ml (BrainVTA, Wuhan, China) was injected into the basal lateral amygdala (BLA) (AP: −1.58 mm; ML: 3.00 mm; DV: −4.50 mm) by a glass micropipette (Sutter Instrument, Novato, CA, USA).

**Fig. 1:**
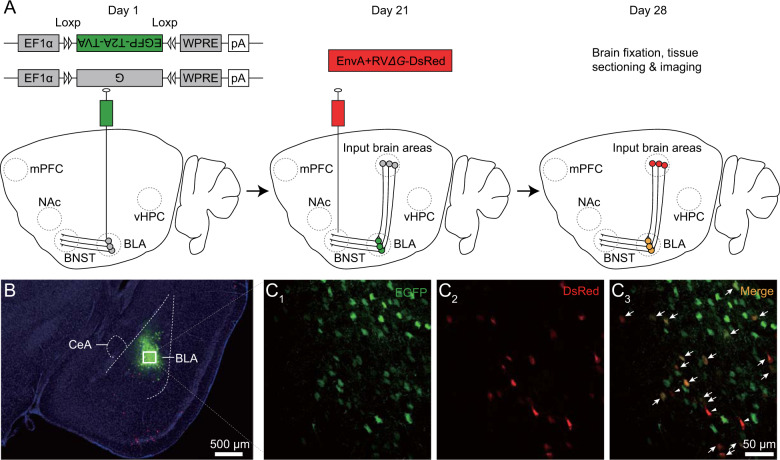
Neuron- and projection-specific retrograde transsynaptic tracing of brain-wide BLA inputs. **A** Schematic of neuron- and projection-specific retrograde transsynaptic tracing brain-wide BLA inputs in the Vglut2-Cre mice. Note that avian ASLV type A protein (EnvA)-pseudotyped glycoprotein (G)-deleted EnvA + RV*∆G*-DsRed rabies viral particles achieve the first presynaptic terminal entry via recombinant TVA receptors, and then G + RV*∆G*-DsRed rabies viral particles budded out with recombinant G proteins on their envelopes achieve the second presynaptic terminal entry via endogenous G protein receptors. Note AAV viral expression of helper genes EGFP-T2A-TVA and G in BLA on day 1, pseudotyped rabies viral expression of EnvA + RV*∆G*-DsRed in BNST, vHPC, mPFC or NAc on day 21, and brain sectioning and imaging of brain-wide monosynaptically connected neurons on day 28. BLA the basolateral complex of the amygdala, BNST the bed nucleus of the stria terminals, mPFC the medial prefrontal cortex, NAc the nucleus accumbens, vHPC the ventral hippocampus. **B** A coronal section of Vglut2-Cre mouse shows AAV and pseudotyped rabies viral co-expression restricted in BLA. **C**_**1–3**_ Enlarged images of boxed area in **B** show starter cells co-expressing EGFP and RV*∆G*-DsRed (green GFP channel, red DsRed channel and overlay). Note yellow starter cells indicated by arrows and red only cells resulted from local transsynaptic spread indicated by arrowheads in **C**_**3**_.

For monosynaptic retrograde tracing, 80 nl of 1:1 mixture of AAV-EF1α-DIO-EGFP-T2A-TVA-hGH-pA and AAV-EF1α-DIO-G-hGH-pA viral solution with the titer of 2 × 10^12^ vg/ml was unilaterally injected into BLA of the Vglut2-Cre and Thy1-Cre mice (AP: −1.58 mm; ML: 3.00 mm; DV: −4.50 mm), which resulted in infection of ~30% glutamatergic neurons in these mice (Figs. 1 and S1, 2). Three weeks after expression of these two AAV viral helpers, 100–200 nl pseudotyped EnvA + RV*∆G*-DsRed rabies viral solution with the titer of 2 × 10^8^ IFU/ml was unilaterally injected into either the BNST, vHPC, mPFC, or NAc. In particular, 100 nl viral solution was delivered to BNST (0.30 mm AP, 1.15 mm ML, 4.25 mm DV), 200 nl to two vHPC sites (AP: −3.08 mm; ML: −3.00 mm; DV: −3.60 mm, and AP: −3.08 mm; ML: −3.00 mm, DV: −4.20 mm), two mPFC sites (AP: 2.10 mm, ML: 0.30 mm, DV: −1.75 mm, and AP: 2.10 mm; ML: 0.30 mm; DV: −2.25 mm), and NAc (AP: 1.10 mm; ML: 0.75 mm; DV: −4.60 mm). The brain samples were collected 1 week after pseudotyped rabies viral expression. As controls of specificity, 80 nl of AAV-EF1α-DIO-EGFP-T2A-TVA-hGH-pA viral solution was injected into BLA of the Vglut2-Cre and Thy1-Cre mice, or 80 nl of 1:1 mixture of AAV-EF1α-DIO-EGFP-T2A-TVA-hGH-pA and AAV-EF1α-DIO-G-hGH-pA viral solution virus mixture was injected into BLA of wild-type mice, followed by 100 nl pseudotyped rabies EnvA + RV*∆G*-DsRed viral solution injection in NAc 3 weeks later. Such experiments resulted in no monosynaptically traced cell from BLA neurons, ruling out any non-specific transsynaptic spread (Fig. S3).

**Fig. 2:**
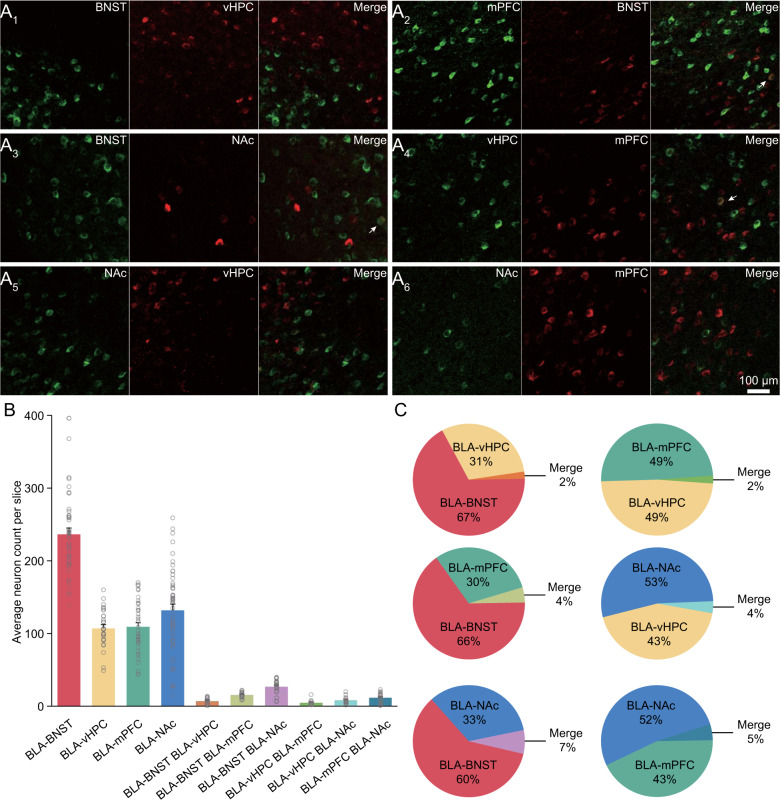
Collateralization analysis of BLA output neuronal groups. **A**_**1–6**_ Coronal tissue section images showing BNST- and vHPC-projecting BLA neurons (**A**_**1**_), mPFC- and BNST-projecting BLA neurons (**A**_**2**_), BNST- and NAc-projecting BLA neurons (**A**_**3**_), vHPC- and mPFC-projecting BLA neurons (**A**_**4**_), NAc- and vHPC-projecting BLA neurons (**A**_**5**_), and NAc- and mPFC-projecting BLA neurons (**A**_**6**_) labeled with CTB 555 and CTB 647, respectively (green CTB 555 channel, red CTB 647channel, and overlay). Note arrows indicating neurons labeled with both CTB 555 and CTB 647. **B** Average numbers of BLA projection neurons labeled with one or two CTB tracers (*n* = 23,876 cells counted in 84 slices prepared from 24 animals). **C** Sector diagrams illustrating percentages of BLA projection neurons labeled with one or two CTB tracers.

### CTB retrograde tracing

CTB 555 and CTB 647 (ThermoFisher Scientific, Waltham, MA, USA) were dissolved in phosphate-buffered saline (PBS) at the concentration of 1.0 mg/ml. To label BNST-, vHPC-, mPFC- and NAc-projecting BLA neurons, we injected CTB 555 and CTB 647 in all two combinations of the four projecting targets in wild-type mice. In particular, 80 nl CTB solution was injected to BNST (0.30 mm AP, 1.15 mm ML, 4.25 mm DV), 120 nl to two vHPC sites (AP: −3.08 mm; ML: −3.00 mm; DV: −3.60 mm, and AP: −3.08 mm; ML: −3.00 mm, DV: −4.20 mm), 120 nl to two mPFC sites (AP: 2.10 mm, ML: 0.30 mm, DV: −1.75 mm, and AP: 2.10 mm; ML: 0.30 mm; DV: −2.25 mm), and 80 nl to NAc (AP: 1.10 mm; ML: 0.75 mm; DV: −4.60 mm). The brain samples were collected 1 week after surgery.

### Tissue preparation

Animal tissue preparation followed the procedure reported in our recent study [10]. Briefly, animals were deeply anesthetized with sodium pentobarbital, immediately perfused with 15 ml of 1× PBS at pH 7.2–7.4 diluted from 10× PBS (Coolaber, Beijing, China), and 15 ml 4% paraformaldehyde (Biosharp, Beijing, China). After perfusion, the brain was carefully removed, post-fixed in 4% PFA overnight at 4 °C, and then dehydrated in 30% sucrose for 48 h. After fixation, the brain was embedded in the optimum cutting temperature formulation of water-soluble glycols and resins (Sakura Finetek, Torrance, CA, USA), and sectioned serially into 50-µm coronal slices with a cryostat tissue slicer (ThermoFisher Scientific, Waltham, MA, USA). The tissue sections were washed (three times, 10 min each) with 1× PBS, stained with Dapi (Coolaber, Beijing, China) for 20 min, washed again (three times, 10 min each) with 1× PBS, and then coverslipped within 50% (v/v) glycerol in 1× PBS. Immunostaining of GABAergic and glutamatergic neurons were made with rabbit anti-GABA (Sigma-Aldrich, A2052, St. Louis, MO, USA) and anti-glutamate (Sigma-Aldrich, G6642) as the primary antibodies and donkey anti-rabbit Alexa Fluor^®^ 647 (Abcam, ab150075, Cambridge, MA, USA) as the secondary antibody.

### Image acquisition and data analysis

The tissue sections were imaged using a high-throughput VS120 Virtual Slide Microscope (Olympus, Shinjuku City, Tokyo, Japan) with a 10× objective. Some sections were imaged using a Nikon A1R laser scanning confocal microscope (Nikon, Minato City, Tokyo, Japan) with 10×, 20× and 60× objectives.

Images of coronal tissue sections were matched to the Allen Mouse Brain Atlas [11], using the Adobe Illustrator (Adobe, Mountain View, CA, USA). The labeled cells were defined, counted using an algorithm based on Image J (NIH, Bethesda, MD, USA), and assigned to corresponding brain areas base on the Allen Mouse Brain Atlas. Afferent input brain regions were tentatively classified into five major groups (i.e., sensory, integrative, contextual, neuromodulatory, and miscellaneous groups) in reference to the previous work [12–14].

### Statistical analysis

Statistical results were reported as mean ± s.e.m. Animals were randomly assigned into control or experimental groups and investigators were blinded to experimental conditions, and no sample was excluded for analysis. Given the negative correlation between the variation and square root of sample number, *n*, the group sample size is typically set to be ~12–25 to optimize the efficiency and power of statistical tests. Statistical significances of the means (*p* < 0.05; two sided) were determined using Mann-Whitney Rank Sum non-parametric tests, and statistical significances of the linear relationships of two data groups were determined using linear regression *t* tests. The normal distribution and similar variance within each comparison group of data were checked prior to statistical tests. The data that support the findings of this study are available from the corresponding author upon request.

## Results

The amygdala sends efferent signals to multiple brain areas to orchestrate defensive behaviors, and the main amygdalar output pathways seem to go through BNST, vHPC, mPFC and NAc to control initiation, acquisition, evaluation and decision-making of survival behaviors, respectively [1, 6]. The previous studies have made significant effort to identify the sources of synaptic inputs to amygdalar neurons and/or the projection targets of these cells [1, 4, 15, 16]. However, technical and/or design limitations preclude these studies from linking the afferent inputs with projection targets of specific amygdalar neuronal groups. To understand the general input-output relationships of the amygdala, we focused our examination on BLA, the primary amygdalar nucleus that serves not only as the main gatekeeper receiving brain-wide afferent inputs [2, 4], but also as a key output center sending efferent projections to various brain areas [6, 16]. Taking advantage of the exclusive property of glycoprotein-deleted rabies virus [17], we adapted a simplified AAV- and pseudotyped rabies virus-based, cell-type- and projection-specific retrograde transsynaptic tracing method in BLA (Figs. 1A and S1A), which capitalized the strengths of similar earlier approaches [18–22]. We first validated that this method enabled tracing of monosynaptic input neurons to specific output BLA neurons in the Vglut2-Cre (Fig. 1B, C) and Thy1-Cre (Fig. S1B, C) mice. We noticed a few red only BLA neurons that displayed only RV*∆G*-DsRed, but not green EGFP fluorescence (Figs. 1C and S1C), suggesting a possible spread of RV*∆G*-DsRed via local circuits. Co-immunostaining analysis revealed that the red only BLA neurons consisted predominately of GABAergic neurons with negligible amount of glutamatergic neurons (~0.05%) displaying only red RV*∆G*-DsRed fluorescence in both the Vglut2-Cre and Thy1-Cre preparations (Fig. S2), indicating minimal multiple synaptic crossing of RV*∆G*-DsRed among glutamatergic neurons in BLA. These results indicate that our simplified tracing method to be highly specific in mapping monosynaptic inputs to particular glutamatergic output neuronal groups in BLA.

We then validated major output pathways of BLA by expressing rAAV-EF1α-DIO-EYFP-WPRE-pA in BLA of the Vglut2-Cre and Thy1-Cre mice. Three weeks after expression, we made brain-wide anterograde axon tracing of output BLA neuronal axons, and our image data validated that the main BLA neuronal axons projected to BNST, vHPC, mPFC and NAc (Fig. S4), consistent with recent reports [6, 16]. A recent analysis indicates that vHPC- and NAc-projecting BLA neurons represent largely independent output neuronal groups [23]. We systematically analyzed the collateralization of BNST-, vHPC-, mPFC- and NAc-projecting BLA neurons with retrograde tracer injections of CTB 555 and CTB 647 in all two combinations of the four targeting areas (Fig. 2). Our results showed that ~5% output BLA neurons sent collateralizations to two targeting areas (Fig. 2B, C), suggesting output BNST-, vHPC-, mPFC- and NAc-projecting BLA neurons to be largely independent groups with small numbers of neurons projecting to multiple brain areas.

### Afferent inputs of the amygdala

**Fig. 3:**
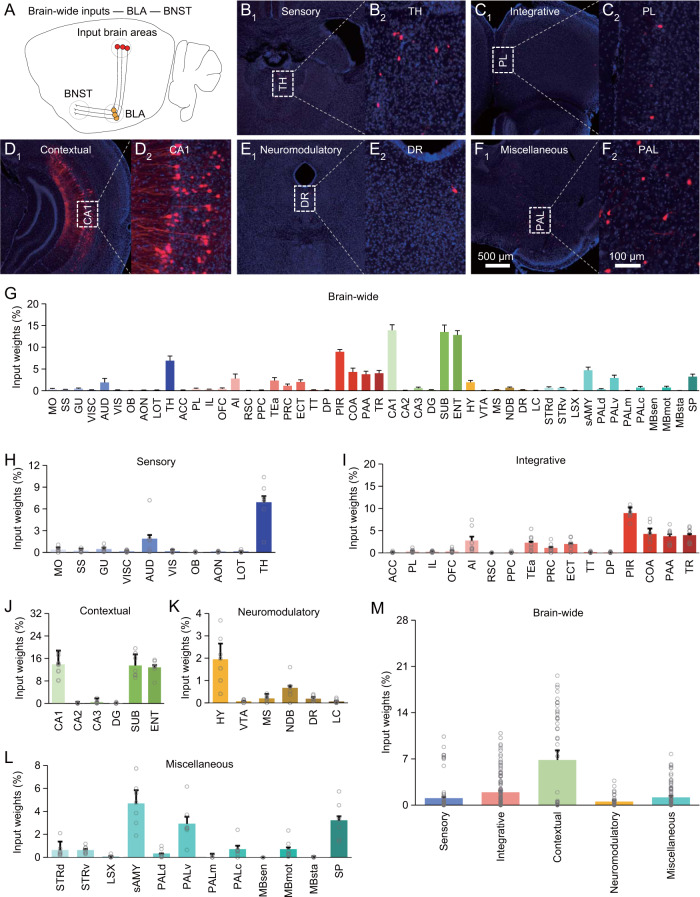
Brain-wide distribution of monosynaptic inputs to Vglut2^+^ BNST-projecting BLA neurons. **A** Schematic of BNST-projecting neuron-specific retrograde transsynaptic tracing in the Vglut2-Cre mice. **B-F**_**1–2**_ Images show cells monosynaptically traced from BNST-projecting BLA neurons back into the sensory (**B**_**1–2**_), integrative (**C**_**1–2**_), contextual (**D**_**1–2**_), neuromodulatory (**E**_**1–2**_) and other miscellaneous (**F**_**1–2**_) brain areas. **G** Percentages of labeled input cells in 50 brain areas (*n* = 21,286 cells from 7 animals). **H** Percentages of labeled input cells carrying sensory stimuli (*n* = 2,444 cells from 7 animals). **I** Percentages of labeled input cells carrying integrative stimuli (*n* = 6,562 cells from 7 animals). **J** Percentages of labeled input cells carrying contextual stimuli (*n* = 8,464 cells from 7 animals). **K** Percentages of labeled input cells carrying neuromodulatory stimuli (*n* = 722 cells from 7 animals). **L** Percentages of labeled input cells carrying other miscellaneous stimuli (*n* = 3,094 cells from 7 animals). **M** Relative input weights of sensory (1.06 ± 0.28 %, *n* = 70 groups from 7 animals), integrative (1.94 ± 0.25 %, *n* = 112 groups from 7 animals), contextual (6.84 ± 1.09 %, *n* = 42 groups from 7 animals), neuromodulatory (0.53 ± 0.13 %, *n* = 42 groups from 7 animals) and other miscellaneous (1.17 ± 0.19 %, *n* = 84 groups from 7 animals) stimuli. See Tables S1 and S2 for statistics.

To investigate the input-output organization of the amygdala, we first utilized the simplified cell-type- and projection-specific retrograde transsynaptic tracing technique to map brain-wide afferent inputs on BLA output neurons projecting to BNST, which is involved in initiating and sustaining anxiety responses [24, 25]. In particular, we made viral expression of AAV-EF1α-DIO-EGFP-T2A-TVA-hGH-pA and AAV-EF1α-DIO-G-hGH-pA in BLA, and 3 weeks later, pseudotyped rabies viral expression of EnvA + RV*∆G*-DsRed in BNST of the Vglut2-Cre mice. After an additional week of expression, we isolated and sectioned the entire mouse brain, and imaged brain-wide areas with monosynaptic afferent inputs to Vglut2 positive (Vglut2^+^) BNST-projecting BLA neurons (Fig. 3A). Brain-wide tissue images showed that a large number of brain areas had neurons forming monosynaptic connections on BNST-projecting BLA neurons (Fig. 3B–G). Since these brain areas are involved in various functions, the data suggest that BNST-projecting BLA neurons receive a variety of afferent stimuli, including sensory, integrative, contextual, neuromodulatory and other miscellaneous inputs (Fig. 3B–G). We repeated the same experiment using the Thy1-Cre mice, and found that Thy1 positive (Thy1^+^) BNST-projecting BLA neurons received similar afferents (Fig. S5A–G). Quantitative analysis revealed that the distinct afferents of Vglut2^+^ and Thy1^+^ BNST-projecting BLA neurons to have varied input weights, with unexpected high representation of contextual inputs, and relatively smaller and similar representations of all other inputs (Figs. 3H–M and S5H–M).

**Fig. 4:**
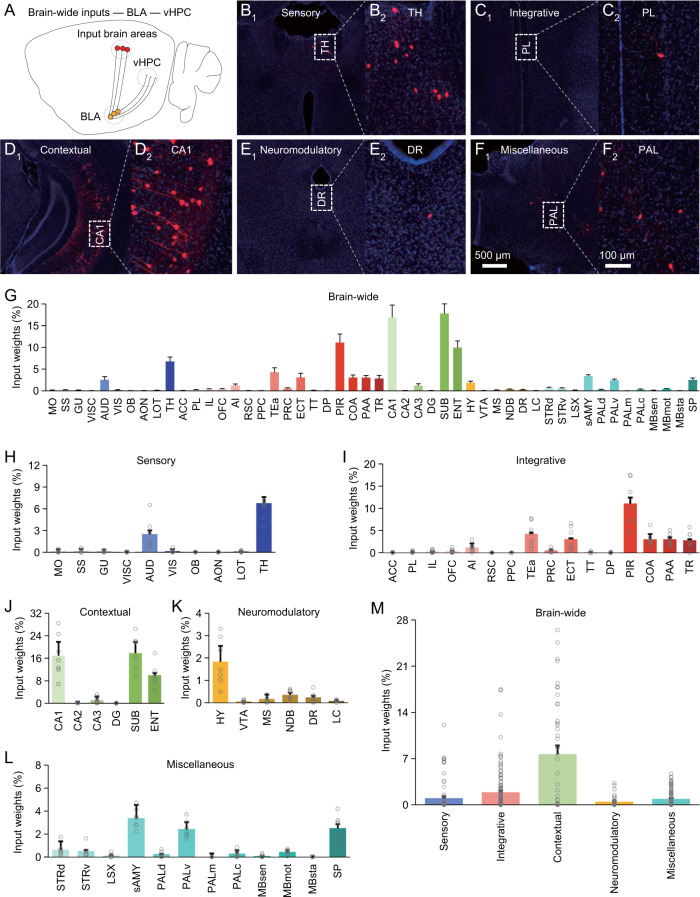
Brain-wide distribution of monosynaptic inputs to Vglut2^+^ vHPC-projecting BLA neurons. **A** Schematic of vHPC-projecting neuron-specific retrograde transsynaptic tracing in the Vglut2-Cre mice. **B-F**_**1–2**_ Images show cells monosynaptically traced from vHPC-projecting BLA neurons back into the sensory (**B**_**1–2**_), integrative (**C**_**1–2**_), contextual (**D**_**1–2**_), neuromodulatory (**E**_**1–2**_) and other miscellaneous (**F**_**1–2**_) brain areas. **G** Percentages of labeled input cells in 50 brain areas (*n* = 22,581 cells from 7 animals). **H** Percentages of labeled input cells carrying sensory stimuli (*n* = 2,495 cells from 7 animals). **I** Percentages of labeled input cells carrying integrative stimuli (*n* = 7,186 cells from 7 animals). **J** Percentages of labeled input cells carrying contextual stimuli (*n* = 9,662 cells from 7 animals). **K** Percentages of labeled input cells carrying neuromodulatory stimuli (*n* = 665 cells from 7 animals). **L** Percentages of labeled input cells carrying other miscellaneous stimuli (*n* = 2,573 cells from 7 animals). **M** Relative input weights of sensory (1.01 ± 0.27 %, *n* = 70 groups from 7 animals), integrative (1.90 ± 0.31 %, *n* = 112 groups from 7 animals), contextual (7.66 ± 1.34 %, *n* = 42 groups from 7 animals), neuromodulatory (0.46 ± 0.12 %, *n* = 42 groups from 7 animals) and other miscellaneous (0.90 ± 0.13 %, *n* = 84 groups from 7 animals) stimuli. See Tables S1 and S2 for statistics.

Next, using the same approach, we examined afferent inputs of BLA output neurons projecting to vHPC (Figs. 4A and S6A), which are necessary for acquisition and modification of fear memory [6, 26]. Counting monosynaptically traced neurons in brain-wide areas showed that Vglut2^+^ vHPC-projecting BLA neurons received a heterogeneous array of afferent stimuli with high input weight for contextual stimuli and low input weights for sensory, integrative, neuromodulatory and other miscellaneous stimuli (Fig. 4B–M). Likewise, Thy1^+^ vHPC-projecting BLA neurons mice had similar afferent inputs (Fig. S6B–M). Together, these results confirm that BLA output neurons receive diverse afferent stimuli with varied input weights.

**Fig. 5:**
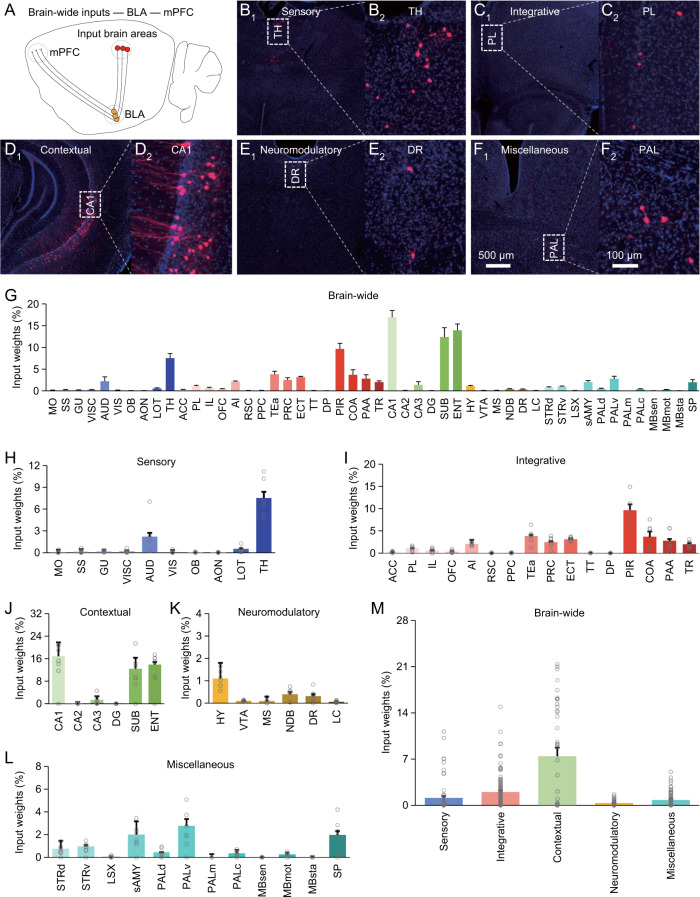
Brain-wide distribution of monosynaptic inputs to Vglut2^+^ mPFC-projecting BLA neurons. **A** Schematic of mPFC-projecting neuron-specific retrograde transsynaptic tracing in the Vglut2-Cre mice. **B-F**_**1–2**_ Images show cells monosynaptically traced from mPFC-projecting BLA neurons back into the sensory (**B**_**1–2**_), integrative (**C**_**1–2**_), contextual (**D**_**1–2**_), neuromodulatory (**E**_**1–2**_) and other miscellaneous (**F**_**1–2**_) brain areas. **G** Percentages of labeled input cells in 50 brain areas (*n* = 12,009 cells from 6 animals). **H** Percentages of labeled input cells carrying sensory stimuli (*n* = 1,289 cells from 6 animals). **I** Percentages of labeled input cells carrying integrative stimuli (*n* = 3,692 cells from 6 animals). **J** Percentages of labeled input cells carrying contextual stimuli (*n* = 5,594 cells from 6 animals). **K** Percentages of labeled input cells carrying neuromodulatory stimuli (*n* = 239 cells from 6 animals). **L** Percentages of labeled input cells carrying other miscellaneous stimuli (*n* = 1,195 cells from 6 animals). **M** Relative input weights of sensory (1.12 ± 0.32 %, *n* = 60 groups from 6 animals), integrative (2.02 ± 0.27 %, *n* = 96 groups from 6 animals), contextual (7.46 ± 1.29 %, *n* = 36 groups from 6 animals), neuromodulatory (0.35 ± 0.07 %, *n* = 36 groups from 6 animals) and other miscellaneous (0.81 ± 0.13 %, *n* = 72 groups from 6 animals) stimuli. See Tables S1 and S2 for statistics.

We then analyzed afferent inputs of BLA output neurons projecting to mPFC (Figs. 5A and S7A), a brain area critical for evaluation and interpretation of fear and anxiety [27–29]. Quantifying monosynaptically traced neurons in brain-wide areas showed that Vglut2^+^ mPFC-projecting BLA neurons received inputs from contextual brain areas with high input weight and sensory, integrative, neuromodulatory and other miscellaneous areas with relatively lower input weights (Fig. 5B–M). Similarly, Thy1^+^ vHPC-projecting BLA neurons received afferents with similar patterns (Fig. S7B–M). Together, these results consistently support the notion that in general BLA output neurons receive diverse afferent stimuli with varied input weights.

**Fig. 6:**
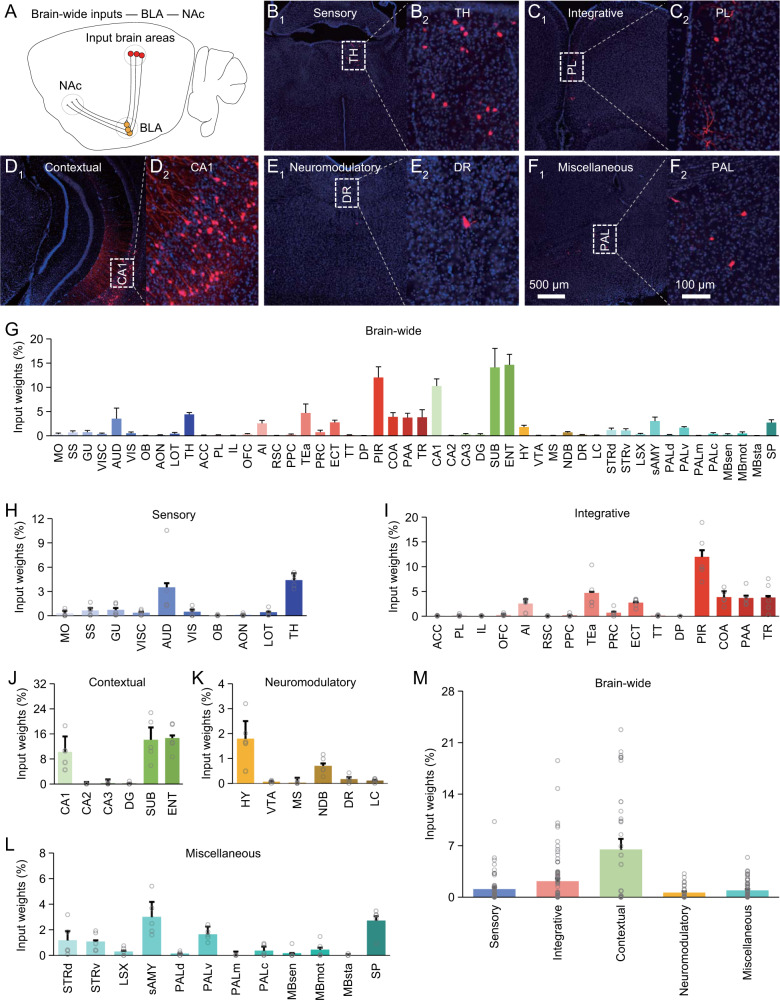
Brain-wide distribution of monosynaptic inputs to Vglut2^+^ NAc-projecting BLA neurons. **A** Schematic of NAc-projecting neuron-specific retrograde transsynaptic tracing in the Vglut2-Cre mice. **B-F**_**1–2**_ Images show cells monosynaptically traced from NAc-projecting BLA neurons back into the sensory (**B**_**1–2**_), integrative (**C**_**1–2**_), contextual (**D**_**1–2**_), neuromodulatory (**E**_**1–2**_) and other miscellaneous (**F**_**1–2**_) brain areas. **G** Percentages of labeled input cells in 50 brain areas (*n* = 10,257 cells from 5 animals). **H** Percentages of labeled input cells carrying sensory stimuli (*n* = 948 cells from 5 animals). **I** Percentages of labeled input cells carrying integrative stimuli (*n* = 3,389 cells from 5 animals). **J** Percentages of labeled input cells carrying contextual stimuli (*n* = 4,489 cells from 5 animals). **K** Percentages of labeled input cells carrying neuromodulatory stimuli (*n* = 308 cells from 5 animals). **L** Percentages of labeled input cells carrying other miscellaneous stimuli (*n* = 1,123 cells from 5 animals). **M** Relative input weights of sensory (1.11 ± 0.27 %, *n* = 50 groups from 5 animals), integrative (2.20 ± 0.39 %, *n* = 80 groups from 5 animals), contextual (6.59 ± 1.39 %, *n* = 30 groups from 5 animals), neuromodulatory (0.48 ± 0.14 %, *n* = 30 groups from 5 animals) and other miscellaneous (0.94 ± 0.16 %, *n* = 60 groups from 5 animals) stimuli. See Tables S1 and S2 for statistics.

Finally, we quantified afferent inputs of BLA output neurons projecting to NAc (Figs. 6A and S8A), a brain area essential for decision-making and initiation of defensive behaviors [30–32]. Calculating monosynaptically traced neurons in brain-wide areas showed that Vglut2^+^ NAc-projecting BLA neurons had inputs from contextual brain areas with high input weight and sensory, integrative, neuromodulatory and other miscellaneous areas with relatively lower input weights (Fig. 6B–M). Re-examination of vHPC-projecting BLA neurons in the Thy1-cre mice revealed similar afferent patterns (Fig. S8B–M). These results verify the general conclusion that BLA output neurons receive diverse afferent stimuli with varied input weights.

### Efferent outputs of the amygdala

**Fig. 7:**
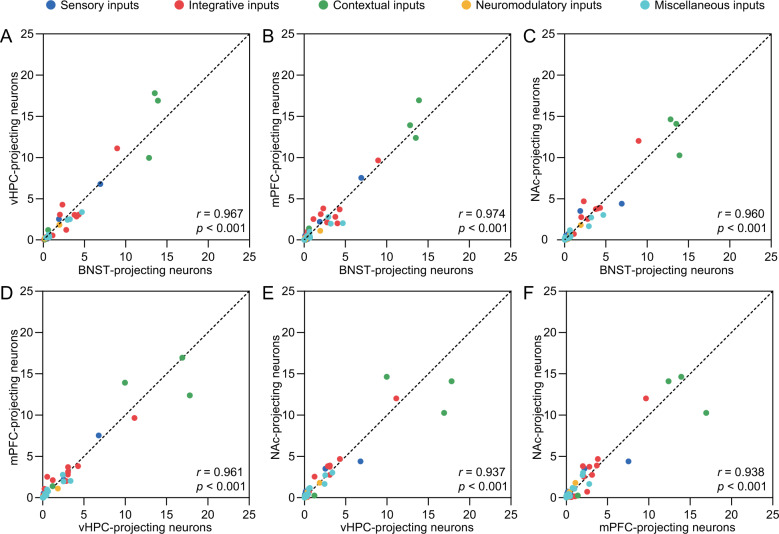
Nearly identical inputs for distinct Vglut2^+^ BLA output neuronal groups. **A**–**F** Scatter plots reveal comparable input weights between Vglut2^+^ BNST-, vHPC-, mPFC- and NAc-projecting BLA neurons. Colored dots represent the average percentage of sensory (cyan), integrative (red), contextual (green), neuromodulatory (orange) and other miscellaneous (blue) inputs. Dashed lines indicate the line of equality. Statistic test values for BNST vs. vHPC (*n* = 50; Normality test: *p* < 0.001; Constant variance test: *p* < 0.001; *r* = 0.967, *p* < 0.001), BNST vs. mPFC (*n* = 50; Normality test: *p* < 0.001; Constant variance test: *p* < 0.001; *r* = 0.974, *p* < 0.001), BNST vs. NAc (*n* = 50; Normality test: *p* < 0.001; Constant variance test: *p* < 0.001; *r* = 0.960, *p* < 0.001), vHPC vs. mPFC (*n* = 50; Normality test: *p* < 0.001; Constant variance test: *p* < 0.001; *r* = 0.961, *p* < 0.001), vHPC vs. NAc (*n* = 50; Normality test: *p* < 0.001; Constant variance test: *p* < 0.001; *r* = 0.937, *p* < 0.001) and mPFC vs. NAc (*n* = 50; Normality test: *p* < 0.001; Constant variance test: *p* < 0.001; *r* = 0.938, *p* < 0.001).

We noted that BNST-, vHPC-, mPFC- and NAc-projecting BLA neurons all receive a diverse array of afferents with varied input weights and predominant contextual representation (Figs. 3–6 and S5–8, Tables S1–4). Hence, we examined correlations of afferent inputs of these four groups of output BLA neurons in both the Vglut2-Cre and Thy1-Cre mice. The correlation analysis showed that all BLA output neuronal groups receive the same set of afferent inputs with identical input weights (Fig. 7 and S9). These results suggest parallel processing of afferent information by major output BLA neuronal groups.

## Discussion

In this study, we systematically investigated afferent and efferent neuronal connections of BNST-, vHPC-, mPFC- and NAc-projecting BLA neurons with a simplified cell-type- and projection-specific retrograde transsynaptic tracing technique. Our analysis reveals two key principles governing the amygdalar input-output organization: (1) BLA output neurons receive a heterogeneous array of brain-wide afferents with varied input weights and predominant representation of contextual information; and (2) BNST-, vHPC-, mPFC- and NAc-projecting BLA neurons receive brain-wide afferents with almost identical origins and input weights. The data suggest that the afferent neuronal connections of BLA are input-dependent, ideally designed to integrate a wide range of sensory, associative, contextual, neuromodulatory and other miscellaneous signals with different weights. Moreover, the efferent neuronal connections of BLA are output-independent, perfectly structured to form multidimensional pathways to in parallel direct the initiation, acquisition, evaluation and decision-making actions of survival behaviors.

### Biological implication

Survival depends on the individual’s ability to integrate a large array of internal and external stimuli and to produce effective behavioral responses. The amygdala, a conserved brain structure, is essential for both the perception and action of survival behaviors, such as fear, anxiety and defensive behaviors [1, 3, 33]. The new insights of BLA input-output organization shed light on how the amygdala converts input stimuli to output behavioral commands. Accumulating anatomical research, including the most recent studies, has consistently documented that the amygdala receives neural projections essentially from every brain area [1, 4, 15, 16]. However, these studies stop short of defining the relationship of amygdalar input and output neuronal circuits. Therefore, it is still unclear how the amygdala processes such a large diverse array of afferent stimuli [1]. Using a simplified cell-type- and projection-specific retrograde transsynaptic tracing method, we here confirm that a variety of sensory, integrative, contextual, neuromodulatory inputs, as well as other miscellaneous inputs, converge on BLA neurons. More importantly, our quantitative analysis reveals that BLA weighs afferent inputs differently. In particular, BLA seems to collect far more contextual information compared to the others (Figs. 3–6M and S5–8M). These results suggest that contextual stimuli have the most prominent representation in BLA and may thus play a dominant role in formatting fear and anxiety responses than other stimuli. Other afferent inputs, including those from the sensory, integrative, and neuromodulatory brain areas, give small, comparable amounts of afferent inputs to BLA, suggesting modest, similar representations. The remaining miscellaneous inputs on BLA appear to have somewhat more input weights than sensory, integrative and neuromodulatory inputs, albeit this conclusion may require revisit as the inputs are likely classifiable into multiple smaller functional groups each with reduced input weights. Interestingly, we observe almost identical afferent input patterns in all four BNST-, vHPC-, mPFC- and NAc-projecting BLA neuronal groups, as well as in Vglut2^+^ and Thy1^+^ BLA output neurons, which represent general and specific populations of glutamatergic BLA neurons [34]. These results are consistent with the notion that the afferent organization represents a general scheme applicable across to all BLA output neurons.

The amygdala is responsible for initiating multiple actions, including initiation, acquisition, evaluation and decision-making processes, to direct defensive behaviors essential for survival [1, 6]. These processes are primarily mediated by amygdalar afferent neuronal connections to BNST, vHPC, mPFC and NAc, respectively, yet how the processing is coordinated is less clear. A recent report proposes that vHPC- and NAc-projecting BLA neurons are largely independent in channeling output information [23]. Our examination of collateralization of BNST-, vHPC-, mPFC- and NAc-projecting BLA neurons reveals that the majority of these four major groups of BLA output neurons project to single downstream targets, consistent with the anatomical segregation of BNST-, vHPC-, mPFC- and NAc-projecting neurons in BLA [16, 23]. These results are indicative of considerable independence of major BLA output afferent pathways. We note that small proportions (~5%) of BNST-, vHPC-, mPFC- and NAc-projecting BLA neurons project to multiple downstream targets (cf. [23]), which might be essential for orchestrating the initiation, acquisition, evaluation and decision-making actions of defensive behaviors [35]. Moreover, our quantitative anatomical data provide the first evidence indicating that BNST-, vHPC-, mPFC- and NAc-projecting BLA neurons receive brain-wide afferents with virtually identical origins and input weights. These results suggest that BLA employs similar input-output computational processes to direct distinct components of defensive behaviors, supporting a recently proposed theory that the amygdala may integrate various afferent stimuli into multidimensional outputs to govern parallel behavioral actions [1].

In this study, our analysis provides the first anatomical BLA input-output neuronal organization scheme. However, due to methodological limitations, rabies-based tracing approaches might introduce some variations while quantifying, for example, brain-wide afferent input weights under different experimental conditions [17]. Indeed, the results from Fu et al. [15], and those from our Vglut2-Cre and Thy1-Cre mice (Figs. 3–6 and S5–8, Tables S1–4) share the similarity in input weight of general afferent stimulus categories, while differ somewhat in input weight of a few specific afferents. Therefore, it is important to validate the findings with independent anatomical approaches [17, 36]. Interestingly, using independent anatomical tracing methods, a recent study supports our findings regarding to the general afferent stimulus category-dependent input weight variance [16]. In addition, the functional BLA input-output neuronal circuits are likely to be dynamically modulated by a variety of biological and environmental factors, such as (pre- and post-) synaptic plasticity, neuromodulation, behavioral states, development and experience [37–40], which may account for varied adaptive defensive behaviors under physiological and pathological conditions (e.g., see [41, 42]).

Our results validate the applicability of a simplified cell type- and projection-specific retrograde transsynaptic tracing method. Since the introduction of cell-type-specific tracing the relationship between input and output method (a.k.a. cTRIO) [21], investigators have adopted the method and revealed various organizational features of neural circuits throughout the nervous system [43]. The rapid adoption and modifications certainly enhance the power and versatility of cTRIO. Among a few modified cTRIO approaches is the one that combines AAV viral expression of TVA and G protein with pseudotyped rabies viral expression of EnvA and RV*∆G* to achieve cell-type-specific tracing of the input–output relationship in cre mice, albeit the specificity of this simplified tracing method has yet been scrutinized [44]. Here, we validate that the simplified tracing method is easy to employ and powerful in mapping the input-output circuit organization in many brain structures with negligible amount of local connections among glutamatergic neurons, such as BLA (Figs. S2 and 3). Given many brain areas encompass types of neurons with minimal local connections among themselves, we expect a broad applicability of this simplified cell-type- and projection-specific retrograde transsynaptic tracing method.

### Clinical implication

The new understanding of anatomical organization of BLA input-output neuronal connections has immediate clinical implication [45–47]. Since the first virtual reality treatment of acrophobia reported in 1995 [48], the virtual reality exposure therapy has emerged as a favored clinical tool to reduce anxiety symptoms in different anxiety disorders: phobias, post-traumatic stress disorders, panic disorder and agoraphobia, social anxiety disorders, psychological stress and generalized anxiety disorders [49, 50]. The unanswered question is why virtual reality treatment is powerful [50]. Our quantitative anatomical analysis of BLA input-output organization, which indicates a dominant contextual representation in afferent inputs in all types of BLA efferent neurons, gives a cue. Indeed, clinical examinations of healthy human subjects and patients with phobia reported that virtual reality accurately reproduced the physiological and behavioral responses evoked by real stimuli [49, 51]. Given the prominent contextual influence in BLA information integration, the real life-mimic virtual situations are expected to be particular powerful in activating and modifying the amygdalar fear-defense system, explaining why virtual reality exposure therapy is particularly effective in treating anxiety disorders [49]. Interestingly, clinical observations report that minimal cues from other stimuli (e.g., sensory and integrative stimuli) are necessary and sufficient to induce synergistic effects on the mainly visual contextual information-based therapy [49, 50]. Our quantitative delineation of modest sensory, integrative, neuromodulatory and other miscellaneous representations in BLA should help to design ideal virtual reality exposure therapies that combine the optimal amounts of other real and imaginal stimuli. Moreover, our analysis of BLA input-output organization may serve a model for demarcating organizational structures of other behavioral systems, and provide effective assessment and treatment options for a variety of other neurological and psychiatric disorders, including autism, chronic pain, depressions and eating disorders [50].

## Supplementary information

Supplemental material
