# Supplementary material for: Organizational principles of amygdalar input-output neuronal circuits
Source: Mol Psychiatry. 2021 Aug 16;26(12):7118–29. doi: 10.1038/s41380-021-01262-3 (PMC8873025; doi:10.1038/s41380-021-01262-3)
Supplement: Supplementary file 1 — Supplemental material [file 41380_2021_1262_MOESM1_ESM.pdf]

Supporting Information for

Organizational principles of amygdalar input-output neuronal circuits

Running title: Quantitative analysis of BLA in and out circuits

Limeng Huang[1,2,3,4,5], Yiwen Chen[3,4], Sen Jin[6], Li Lin[7], Shumin Duan[3,4,8], Ke Si[2,3,4,8], Wei Gong[1,3,4] and J. Julius Zhu[5]

[1]Department of Neurobiology and Department of Neurology of the Second Affiliated Hospital, Zhejiang University School of Medicine, Hangzhou 310009, China
[2]State Key Lab of Modern Optical Instrumentation, College of Optical Science and Engineering, International Research Center for Advanced Photonics, Zhejiang University, Zhejiang 310027, China
[3]Liangzhu Laboratory, Zhejiang University Medical Center, 1369 West Wenyi Road, Hangzhou 311121, China
[4]MOE Frontier Science Center for Brain Science & Brain-Machine Integration, NHC and CAMS Key Laboratory of Medical Neurobiology, School of Brain Science and Brain Medicine, Zhejiang University, Hangzhou 310058, China
[5]Department of Pharmacology, University of Virginia School of Medicine, Charlottesville, VA 22908
[6]Shenzhen Institutes of Advanced Technology, Chinese Academy of Sciences, Shenzhen 518055, China
[7]School of Pharmaceutical Sciences, Wenzhou Medical University, Wenzhou 325035, China
[8]Research Units for Emotion and Emotion Disorders, Chinese Academy of Medical Sciences, Hangzhou, 310058, China

Figure S1

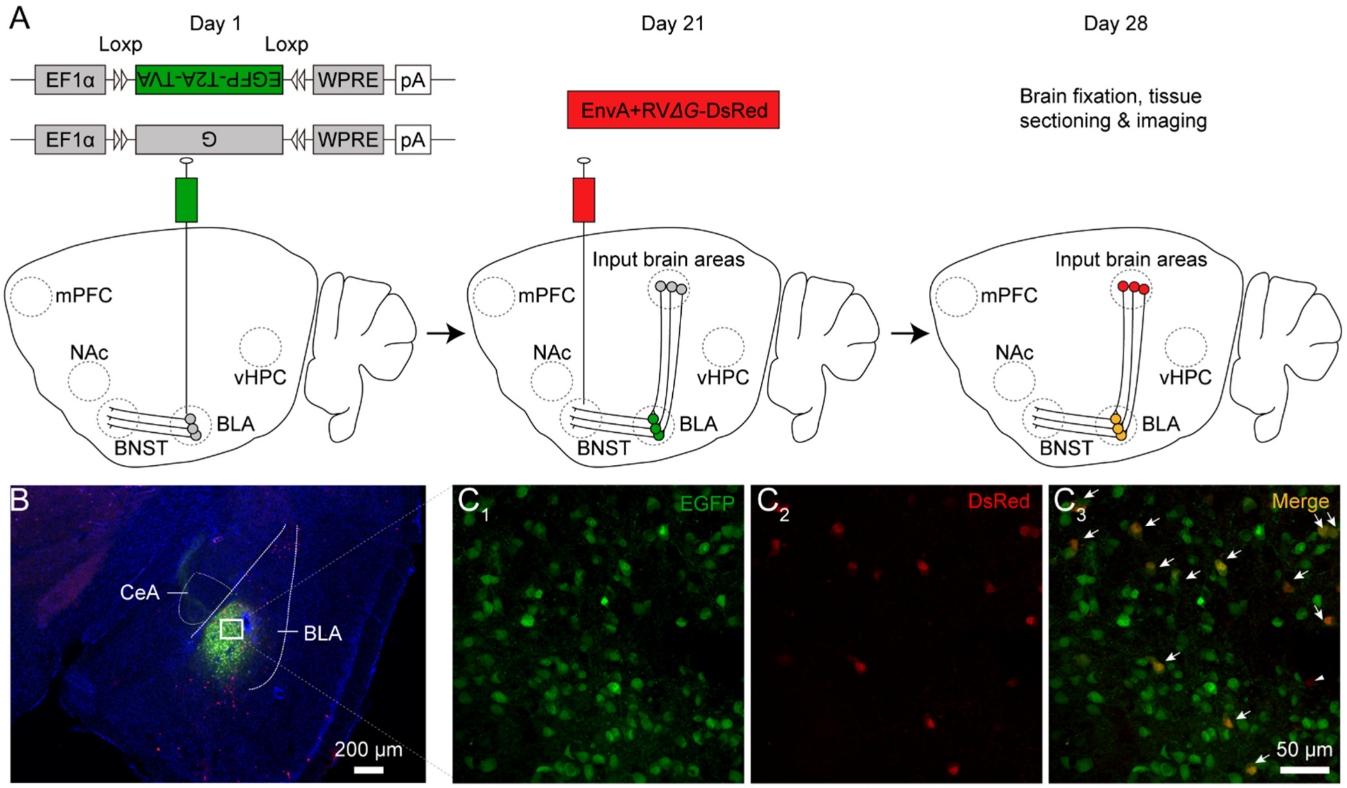

Figure S1. Neuron- and projection-specific retrograde transsynaptic tracing brain-wide BLA inputs.

(**A**) Schematic of neuron- and projection-specific retrograde transsynaptic tracing brain-wide amygdalar inputs in Thy1-Cre mice. Note that avian ASLV type A protein (**EnvA**)-pseudotyped glycoprotein (**G**)-deleted EnvA+RVΔ*G*-DsRed rabies viral particles achieve the first presynaptic terminal entry via recombinant TVA receptors, and then G+RVΔ*G*-DsRed rabies viral particles budded out with recombinant G proteins on their envelopes achieve the second presynaptic terminal entry via endogenous G protein receptors. Note AAV viral expression of helper genes EGFP-T2A-TVA and G in BLA on day 1, pseudotyped rabies viral expression of EnvA+RVΔ*G*-DsRed in BNST, vHPC, mPFC or NAc on day 21, and brain sectioning and imaging of brain-wide monosynaptically connected neurons on day 28. BLA: the basolateral complex of the amygdala, BNST: the bed nucleus of the stria terminals, mPFC: the medial prefrontal cortex, NAc: the nucleus accumbens, vHPC: the ventral hippocampus.

(**B**) A coronal section of Thy1-Cre mouse shows AAV and pseudotyped rabies viral co-expression restricted in BLA.

(**C**$_{1\text{-}3}$) Enlarged images of boxed area in **B** show starter cells co-expressing EGFP and RV∆*G*-DsRed (green GFP channel, red DsRed channel and overlay). Note yellow starter cells indicated by arrows and red only cell resulted from local transsynaptic spread indicated by arrowhead in **C**$_3$.

Figure S2

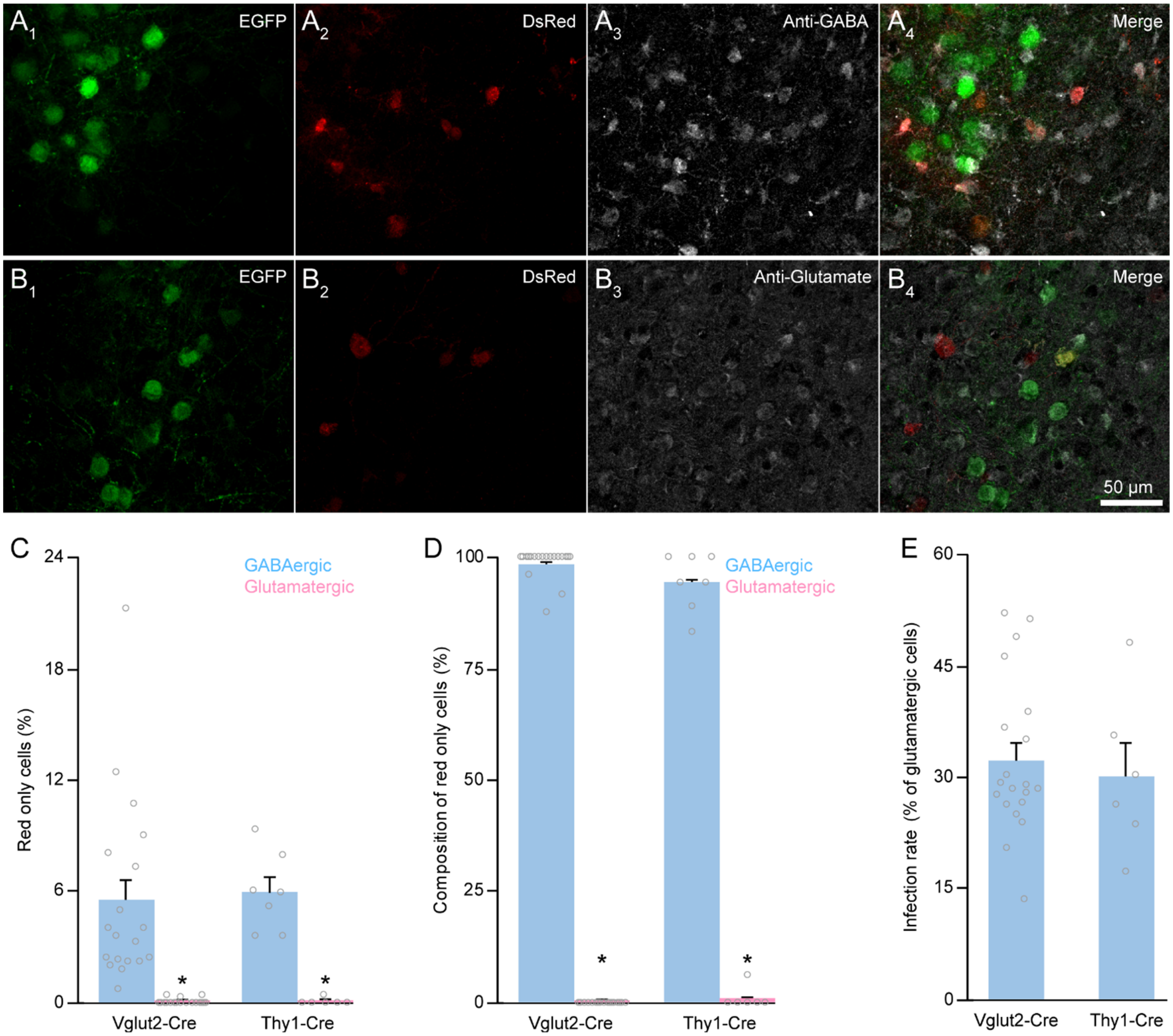

Figure S2. Negligible spread of RVΔ*G*-DsRed among BLA glutamatergic neurons.

(**A**) Co-immunostaining images show RVΔ*G*-DsRed expressing BLA neurons to be GABAergic. Note all RVΔ*G*-DsRed expressing GABAergic BLA neurons, indicated by arrows in **A$_4$**, expressing no EGFP.

(**B**) Co-immunostaining images show RVΔ*G*-DsRed expressing glutamatergic neuron co-expressing EGFP. Note no glutamatergic BLA neurons expressing RVΔ*G*-DsRed only (without EGFP) in **B$_4$**.

(**C**) Percentages of red only BLA neurons (that displayed only RVΔ*G*-DsRed, but not green EGFP fluorescence) in GABAergic and glutamatergic BLA neuron populations of Vglut2-Cre (GABAergic: 5.52±1.16 %, n = 19 slices prepared from 9 mice; Glutamatergic: 0.04±0.03 %, n = 20 slices prepared from 8 mice; U = 0.0, $p < 0.001$) and

Thy1-Cre (GABAergic: 5.95±0.80 %, *n* = 7 slices prepared from 3 mice; Glutamatergic: 0.07±0.07 %, *n* = 7 slices prepared from 3 mice; *U* = 42.0, *p* = 0.001) mice.

(**D**) Relative contributions of GABAergic and glutamatergic BLA neuron populations to red only BLA neurons (that displayed only RVΔ*G*-DsRed, but not green EGFP fluorescence) in Vglut2-Cre (GABAergic: 98.68±0.79 %, *n* = 19 slices prepared from 9 mice; Glutamatergic: 0.01±0.00 %, *n* = 20 slices prepared from 8 mice; *U* = 0.0, *p* < 0.001) and Thy1-Cre (GABAergic: 94.40±2.43 %, *n* = 7 slices prepared from 3 mice; Glutamatergic: 1.04% ± 1.04%, *n* = 7 slices prepared from 3 mice; *U* = 42.0, *p* = 0.001) mice. Asterisks indicate *p* < 0.05 (Mann-Whitney test).

(**E**) Co-immunostaining revealed AAV viral infection rates in glutamatergic BLA neurons of Vglut2-Cre and Thy1-Cre mice (Vglut2-Cre: 32.26±2.34 %, *n* = 20 slices prepared from 8 mice; Thy1-Cre: 30.16±4.37 %, *n* = 6 slices prepared from 3 mice; *U* = 69.0, *p* = 0.605).

Figure S3

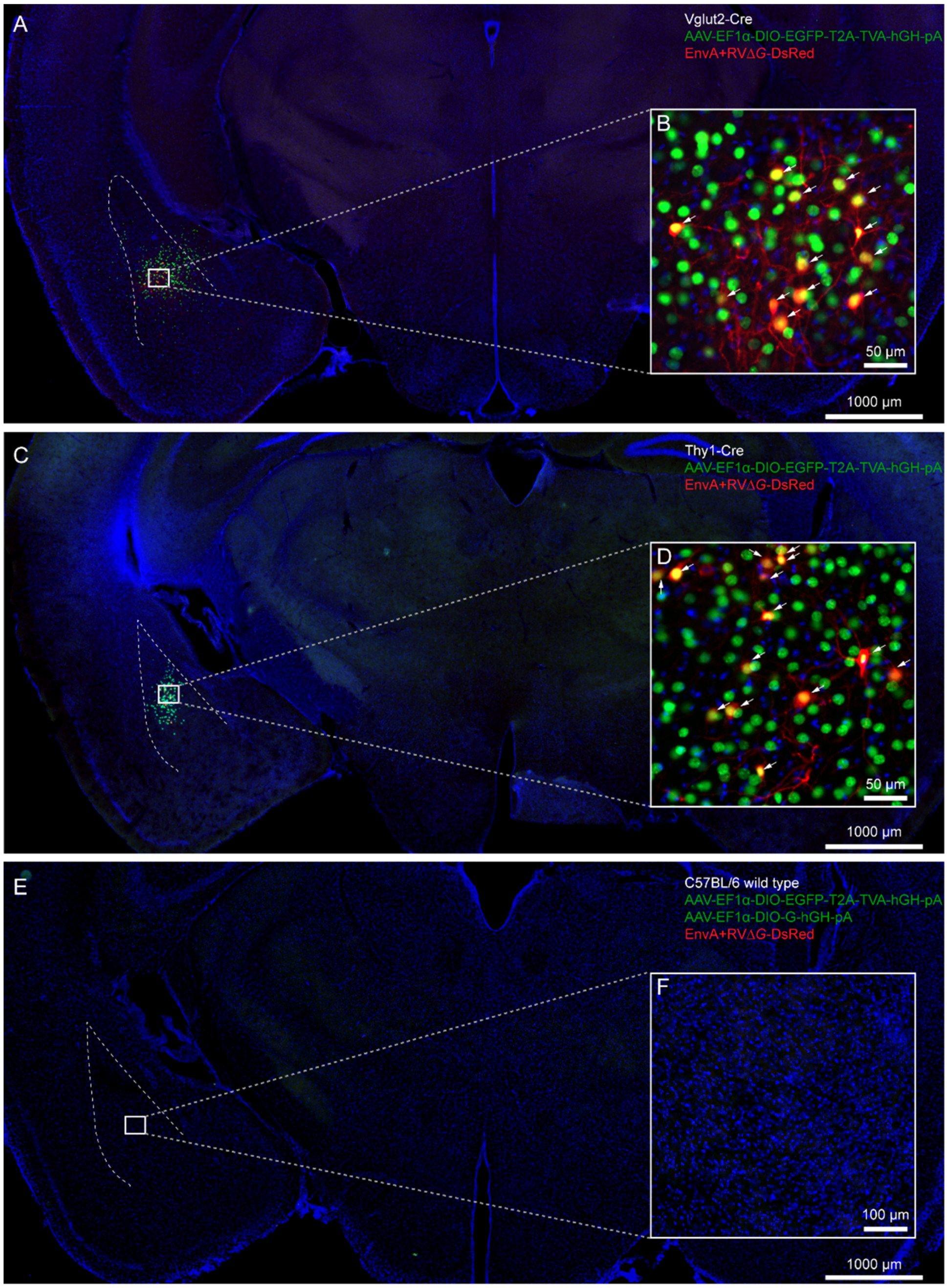

Figure S3. Validation of specificity of retrograde transsynaptic tracing.

(**A-D**) Expression of AAV-EF1α-DIO-EGFP-T2A-TVA-hGH-pA alone in BLA followed by expression of EnvA+RVΔ*G*-DsRed in NAc of the Vglut2-Cre (**A-B**) and Thy1-Cre (**C-D**) mice resulted in EGFP-T2A-TVA (green) expressing cells, and EGFP-T2A-TVA (green) and EnvA+RVΔ*G*-DsRed (red) co-expressing cells in BLA, but no transsynaptic RVΔ*G*-DsRed (red) expressing cells in any brain areas. Note BLA in the boxed area (**A**

and C) enlarged in **B** and **D**, in which EGFP-T2A-TVA (green) and EnvA+RVΔ*G*-DsRed (red) co-expressing cells are indicated by arrows.

(**E-F**) Co-expression of AAV-EF1α-DIO-EGFP-T2A-TVA-hGH-pA and AAV-EF1α-DIO-G-hGH-pA in BLA followed by expression of EnvA+RVΔ*G*-DsRed in NAc of wild type mice resulted in neither EGFP-T2A-TVA (green) and/or EnvA+RVΔ*G*-DsRed (red) expressing cells in BLA, nor transsynaptic RVΔ*G*-DsRed (red) expressing cells in any brain areas.

Figure S4

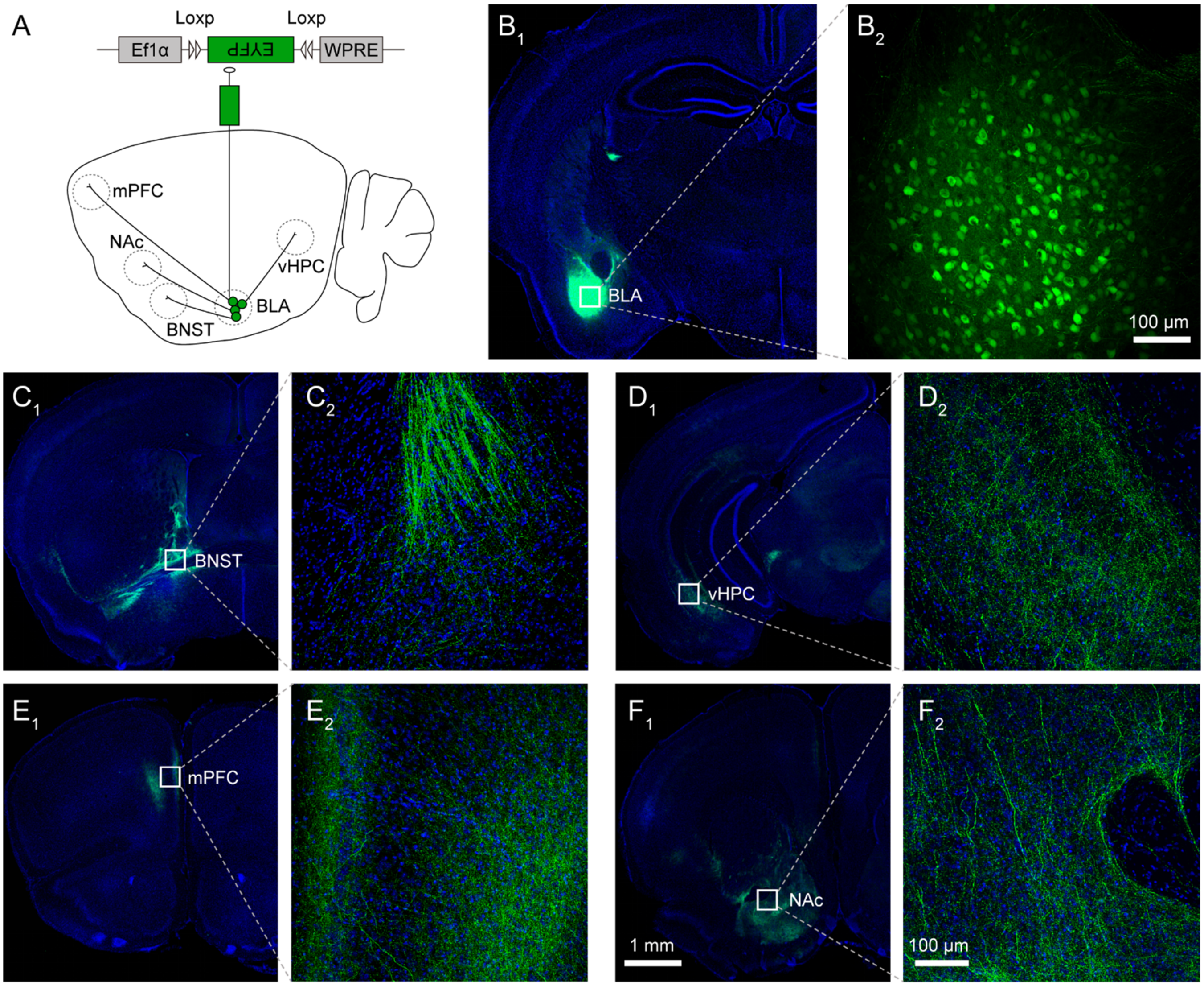

Figure S4. Main output targets of BLA.

(**A**) Schematic of neuron-specific anterograde tracing of BLA outputs in the Vglut2-Cre (n = 6) and Thy1-Cre (n = 4) mice.

(**B_{1-2}**) Images of coronal tissue sections show AAV viral expression in BLA and efferent fibers leaving BLA. Note BLA in the boxed area (**B_1**) enlarged in **B_2**.

(**C-F_{1-2}**) Images of coronal tissue sections show anterograde traced axonal terminals of BLA neurons in BNST, vHPC, mPFC and NAc. Note BNST, vHPC, mPFC and NAc in the boxed areas (**C_1**, **D_1**, **E_1**, and **F_1**) enlarged in **C_2**, **D_2**, **E_2**, and **F_2**, respectively.

Figure S5

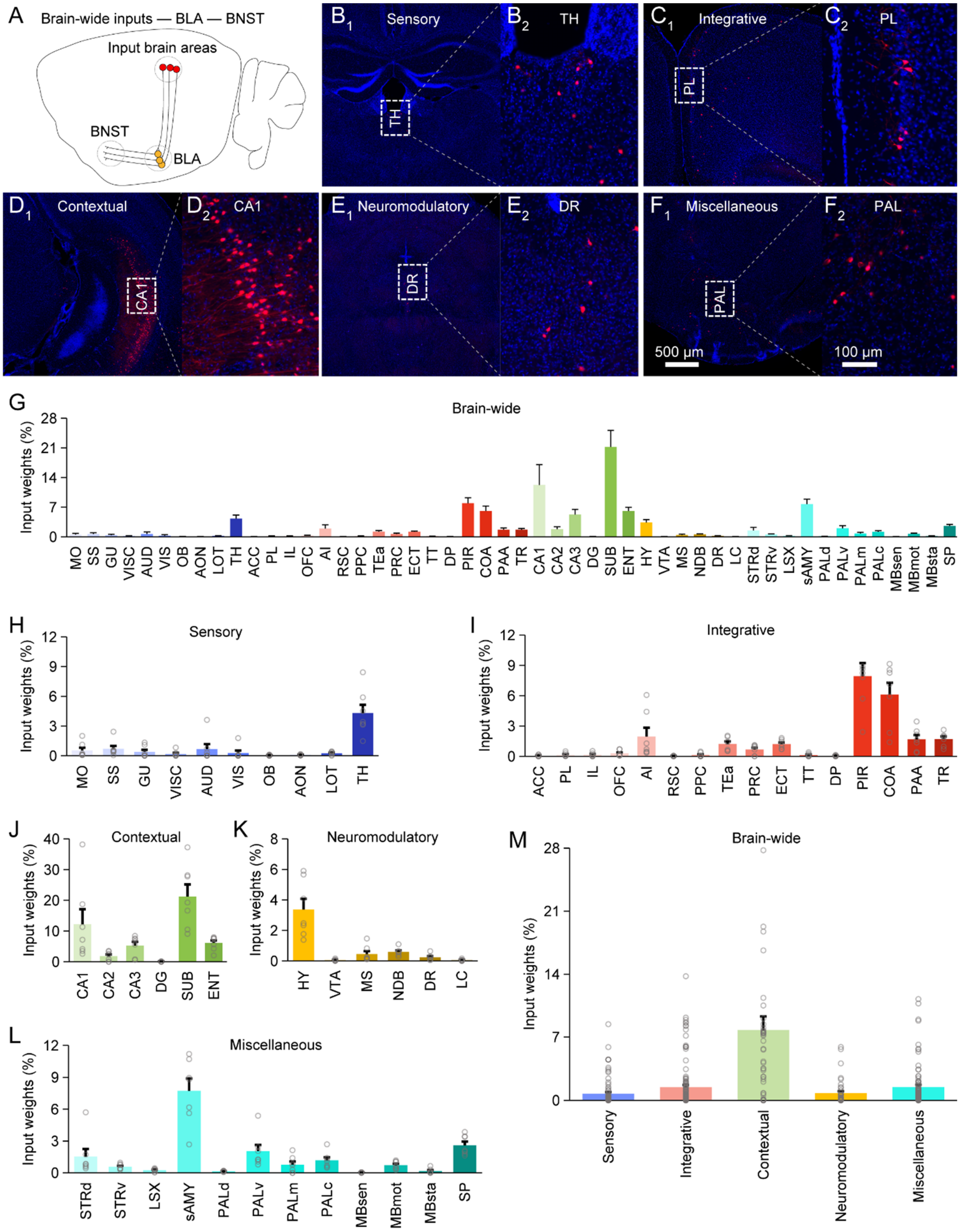

Figure S5. Brain-wide distribution of monosynaptic inputs to Thy1+ BNST-projecting BLA neurons.

(**A**) Schematic of BNST-projecting neuron-specific retrograde transsynaptic tracing in the Thy1-Cre mice.

(**B-F$_{1\text{-}2}$**) Images show cells monosynaptically traced from BNST-projecting BLA neurons back into the sensory (**B$_{1\text{-}2}$**), integrative (**C$_{1\text{-}2}$**), contextual (**D$_{1\text{-}2}$**), neuromodulatory (**E$_{1\text{-}2}$**) and other miscellaneous (**F$_{1\text{-}2}$**) brain areas.

(**G**) Percentages of labeled input cells in 50 brain areas (*n* = 49,935 cells from 7 animals).

(**H**) Percentages of labeled input cells carrying sensory stimuli (*n* = 3,874 cells from 7 animals).

(**I**) Percentages of labeled input cells carrying integrative stimuli (*n* = 11,067 cells from 7 animals).

(**J**) Percentages of labeled input cells carrying contextual stimuli (*n* = 24,307 cells from 7 animals).

(**K**) Percentages of labeled input cells carrying neuromodulatory stimuli (*n* = 2,192 cells from 7 animals).

(**L**) Percentages of labeled input cells carrying other miscellaneous stimuli (*n* = 8,495 cells from 7 animals).

(**M**) Relative input weights of sensory (0.74±0.18 %, *n* = 70 groups from 7 animals), integrative (1.47±0.24 %, *n* = 112 groups from 7 animals), contextual (7.79±1.50 %, *n* = 42 groups from 7 animals), neuromodulatory (0.79±0.22 %, *n* = 42 groups from 7 animals) and other miscellaneous (1.48±0.26 %, *n* = 84 groups from 7 animals) stimuli. See Tables S3 and S4 for statistics.

Figure S6

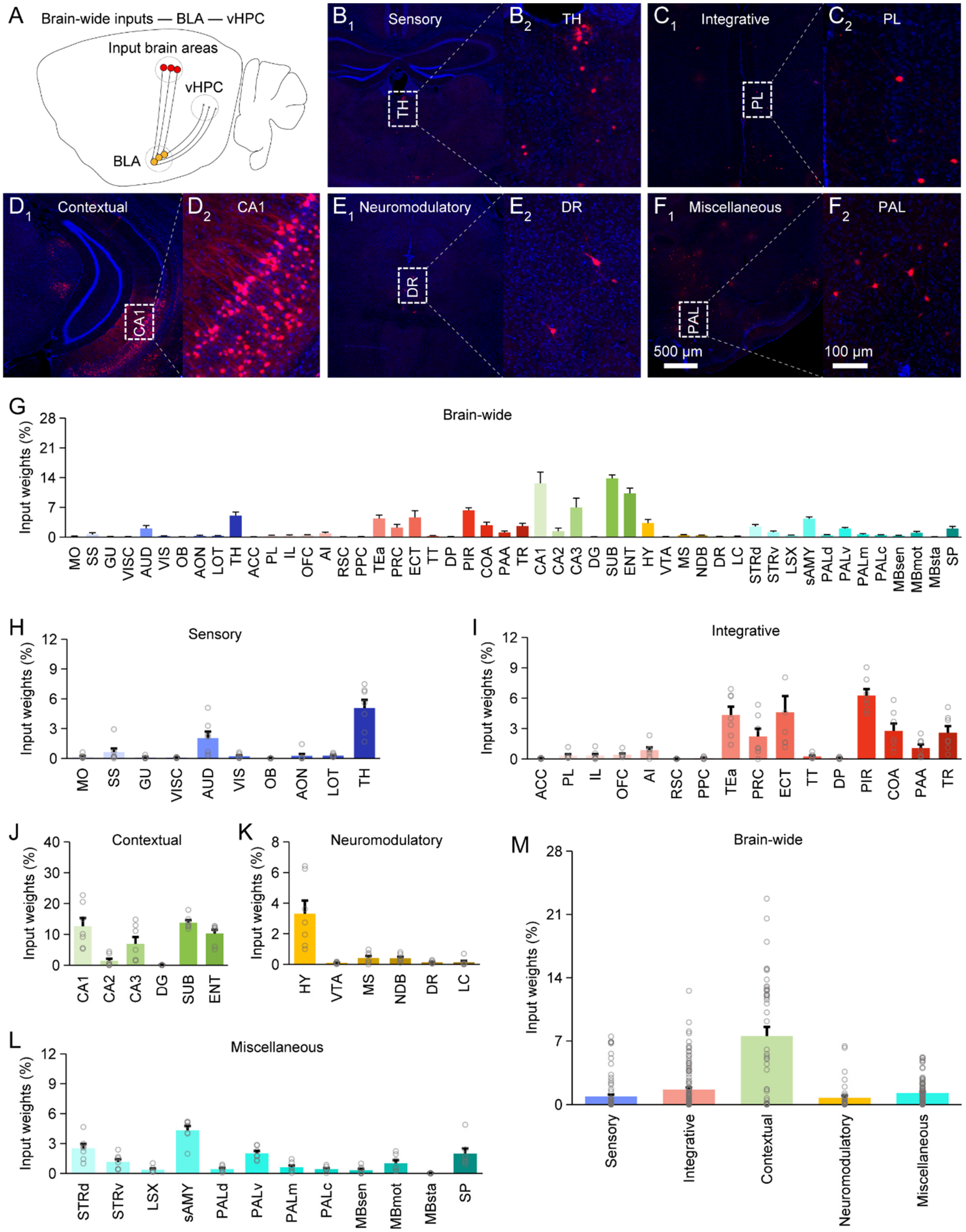

Figure S6. Brain-wide distribution of monosynaptic inputs to Thy1+ vHPC-projecting BLA neurons.

(**A**) Schematic of vHPC-projecting neuron-specific retrograde transsynaptic tracing in the Thy1-Cre mice.

(**B-F$_{1\text{-}2}$**) Images show cells monosynaptically traced from vHPC-projecting BLA neurons back into the sensory (**B$_{1\text{-}2}$**), integrative (**C$_{1\text{-}2}$**), contextual (**D$_{1\text{-}2}$**), neuromodulatory (**E$_{1\text{-}2}$**) and other miscellaneous (**F$_{1\text{-}2}$**) brain areas.

(**G**) Percentages of labeled input cells in 50 brain areas (*n* = 51,057 cells from 7 animals).

(**H**) Percentages of labeled input cells carrying sensory stimuli (*n* = 4,372 cells from 7 animals).

(**I**) Percentages of labeled input cells carrying integrative stimuli (*n* = 14,611 cells from 7 animals).

(**J**) Percentages of labeled input cells carrying contextual stimuli (*n* = 21,791 cells from 7 animals).

(**K**) Percentages of labeled input cells carrying neuromodulatory stimuli (*n* = 2,163 cells from 7 animals).

(**L**) Percentages of labeled input cells carrying other miscellaneous stimuli (*n* = 8,120 cells from 7 animals).

(**M**) Relative input weights of sensory (0.88±0.21 %, *n* = 70 groups from 7 animals), integrative (1.65±0.23 %, *n* = 112 groups from 7 animals), contextual (7.52±1.02 %, *n* = 42 groups from 7 animals), neuromodulatory (0.75±0.23 %, *n* = 42 groups from 7 animals) and other miscellaneous (1.27±0.15 %, *n* = 84 groups from 7 animals) stimuli. See Tables S3 and S4 for statistics.

Figure S7

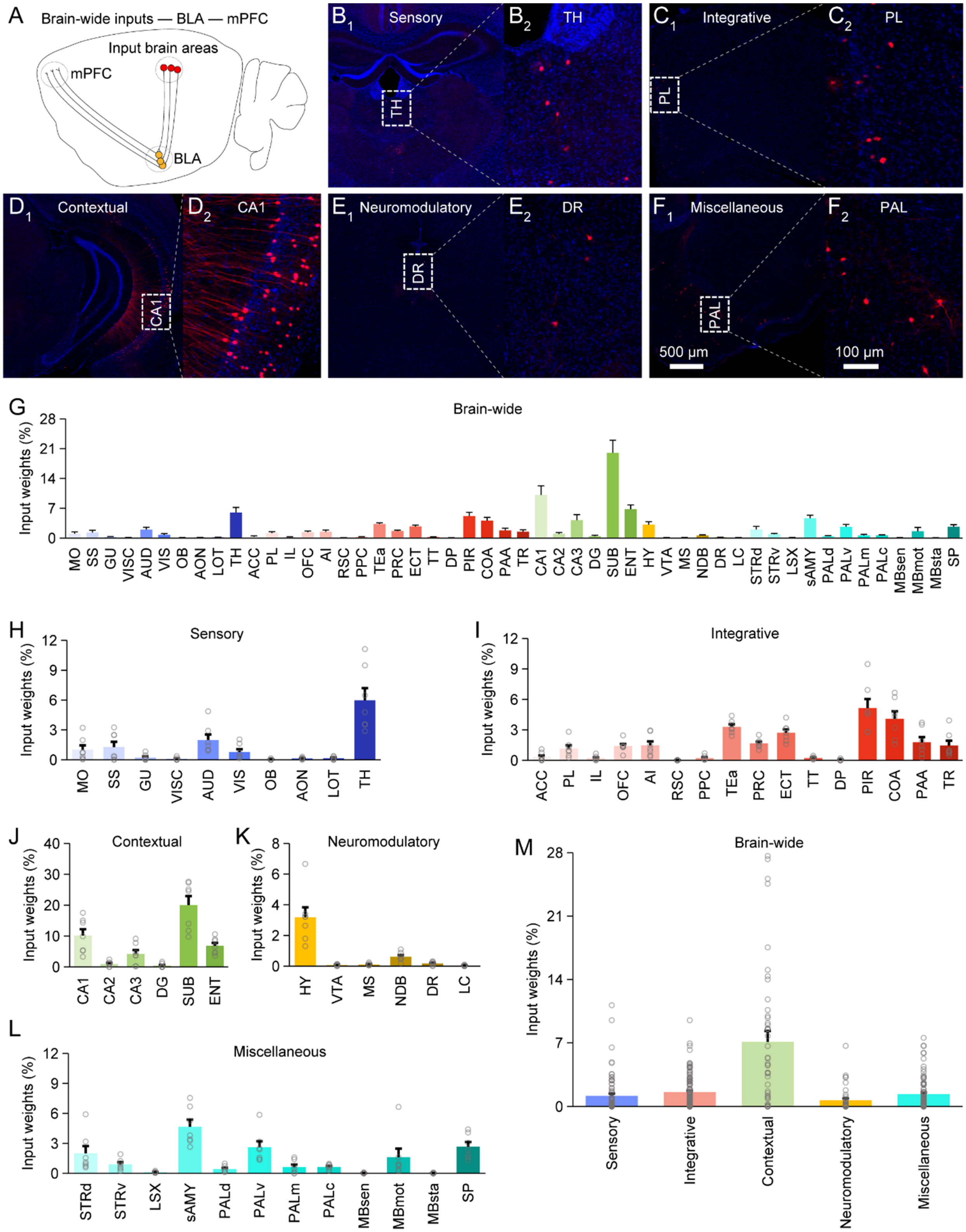

Figure S7. Brain-wide distribution of monosynaptic inputs to Thy1⁺ mPFC-projecting BLA neurons.

(**A**) Schematic of mPFC-projecting neuron-specific retrograde transsynaptic tracing in the Thy1-Cre mice.

(**B-F$_{1-2}$**) Images show cells monosynaptically traced from mPFC-projecting BLA neurons back into the sensory (**B$_{1-2}$**), integrative (**C$_{1-2}$**), contextual (**D$_{1-2}$**), neuromodulatory (**E$_{1-2}$**) and other miscellaneous (**F$_{1-2}$**) brain areas.

(**G**) Percentages of labeled input cells in 50 brain areas (n = 31,345 cells from 7 animals).

(**H**) Percentages of labeled input cells carrying sensory stimuli (n = 3,334 cells from 7 animals).

(**I**) Percentages of labeled input cells carrying integrative stimuli (n = 8,040 cells from 7 animals).

(**J**) Percentages of labeled input cells carrying contextual stimuli (n = 13,974 cells from 7 animals).

(**K**) Percentages of labeled input cells carrying neuromodulatory stimuli (n = 1,287 cells from 7 animals).

(**L**) Percentages of labeled input cells carrying other miscellaneous stimuli (n = 4,710 cells from 7 animals).

(**M**) Relative input weights of sensory (1.17±0.25 %, n = 70 groups from 7 animals), integrative (1.58±0.17 %, n = 112 groups from 7 animals), contextual (7.09±1.22 %, n = 42 groups from 7 animals), neuromodulatory (0.69±0.20 %, n = 42 groups from 7 animals) and other miscellaneous (1.36±0.19 %, n = 84 groups from 7 animals) stimuli. See Tables S3 and S4 for statistics.

Figure S8

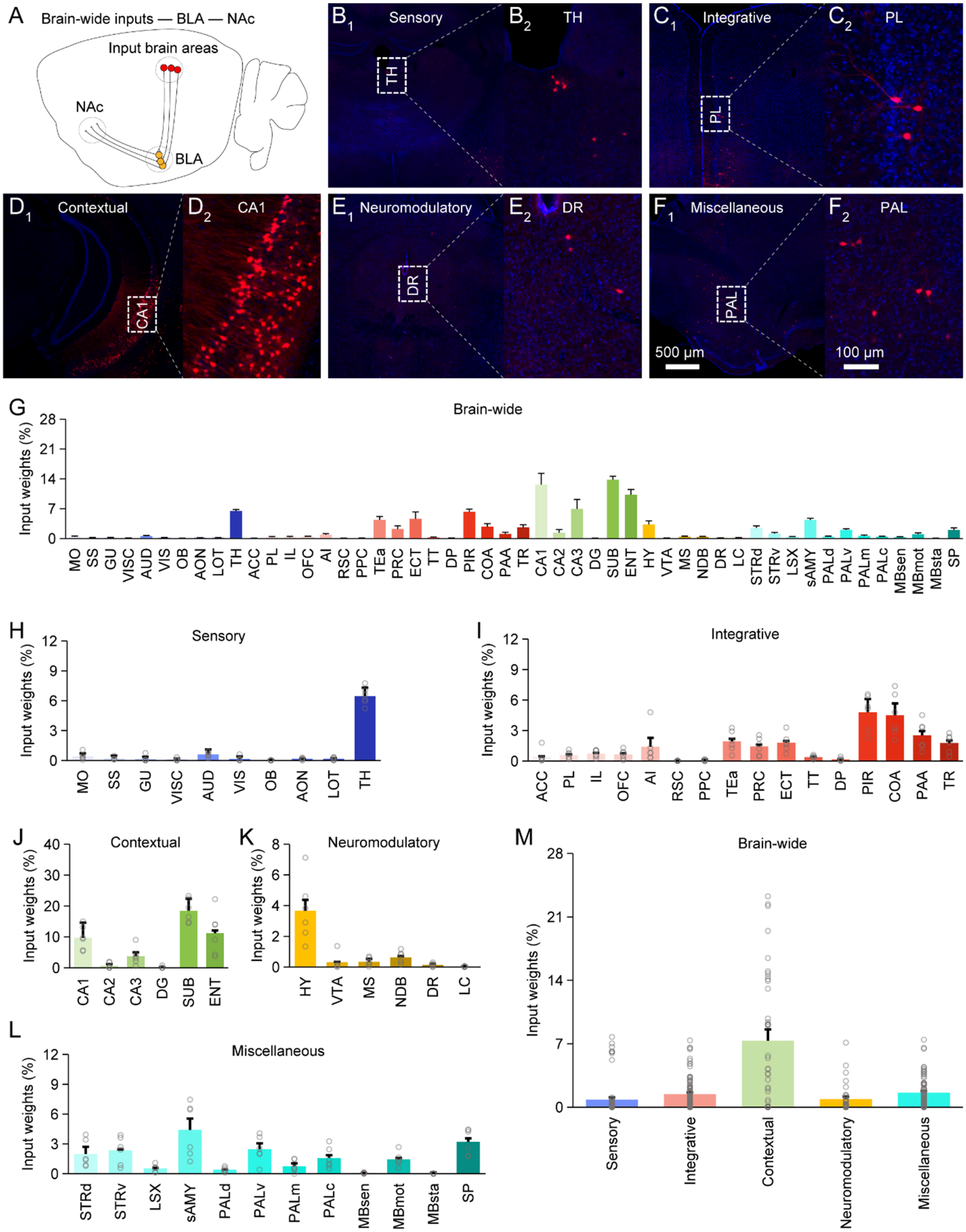

Figure S8. Brain-wide distribution of monosynaptic inputs to Thy1⁺ NAc-projecting BLA neurons.

(**A**) Schematic of NAc-projecting neuron-specific retrograde transsynaptic tracing in the Thy1-Cre mice.

(**B-F$_{1\text{-}2}$**) Images show cells monosynaptically traced from NAc-projecting BLA neurons back into the sensory (**B$_{1\text{-}2}$**), integrative (**C$_{1\text{-}2}$**), contextual (**D$_{1\text{-}2}$**), neuromodulatory (**E$_{1\text{-}2}$**) and other miscellaneous (**F$_{1\text{-}2}$**) brain areas.

(**G**) Percentages of labeled input cells in 50 brain areas (*n* = 28,032 cells from 6 animals).

(**H**) Percentages of labeled input cells carrying sensory stimuli (*n* = 2,378 cells from 6 animals).

(**I**) Percentages of labeled input cells carrying integrative stimuli (*n* = 6,553 cells from 6 animals).

(**J**) Percentages of labeled input cells carrying contextual stimuli (*n* = 12,410 cells from 6 animals).

(**K**) Percentages of labeled input cells carrying neuromodulatory stimuli (*n* = 1,403 cells from 6 animals).

(**L**) Percentages of labeled input cells carrying other miscellaneous stimuli (*n* = 5,288 cells from 6 animals).

(**M**) Relative input weights of sensory (0.84±0.25 %, *n* = 60 groups from 6 animals), integrative (1.45±0.17 %, *n* = 96 groups from 6 animals), contextual (7.34±1.25 %, *n* = 36 groups from 6 animals), neuromodulatory (0.85±0.26 %, *n* = 36 groups from 6 animals) and other miscellaneous (1.60±0.20 %, *n* = 72 groups from 6 animals) stimuli. See Tables S3 and S4 for statistics.

Figure S9

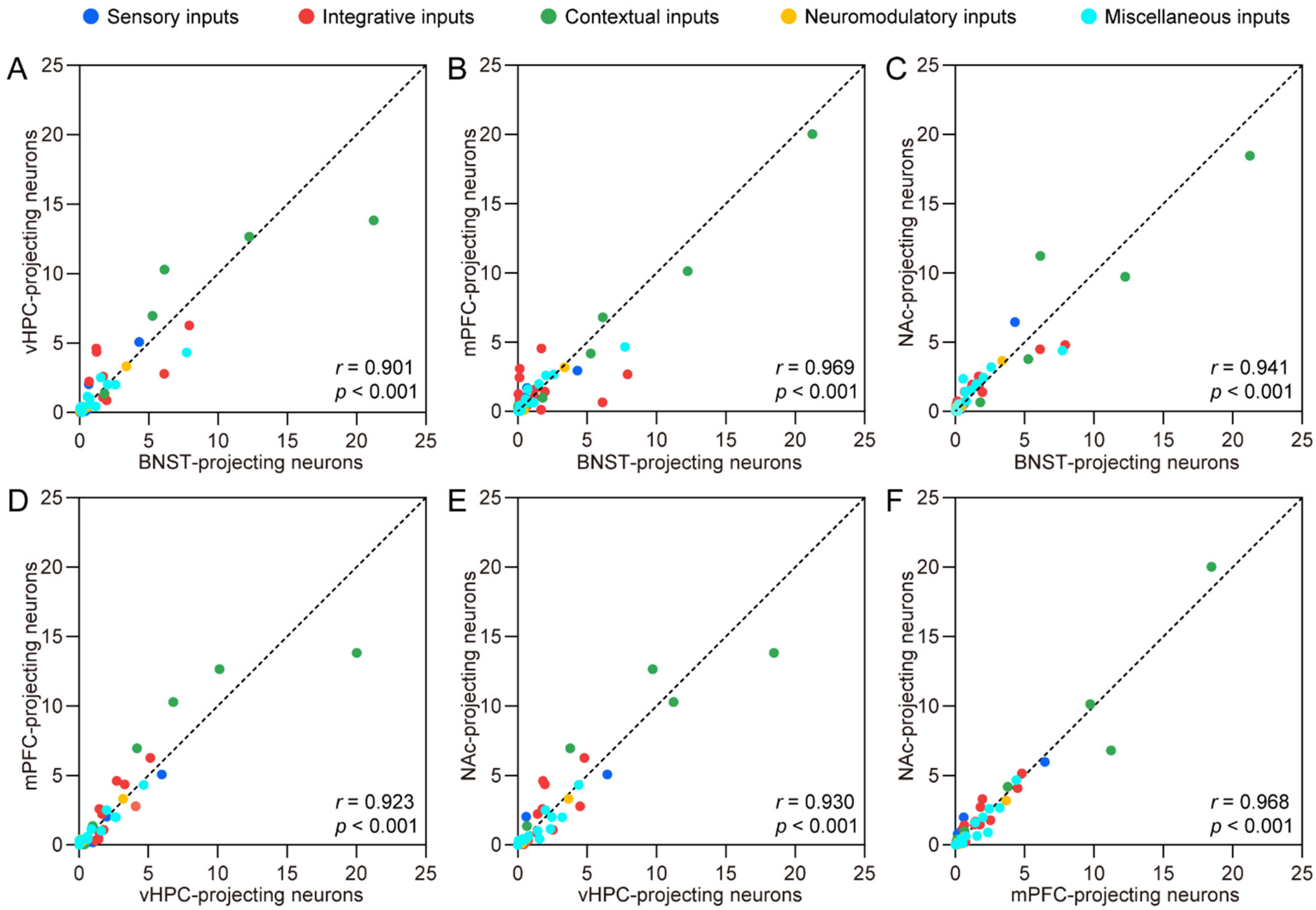

Figure S9. Nearly identical inputs for distinct Thy1⁺ BLA output neuronal groups.

(**A-F**) Scatter plots reveal comparable input weights between Thy1⁺ BNST-, vHPC-, mPFC-, and NAc-projecting BLA neurons. Colored dots represent the average percentage of sensory (cyan), integrative (red), contextual (green), neuromodulatory (orange), and other miscellaneous (blue) inputs. Dashed lines indicate the line of equality. Statistic test values for BNST vs. vHPC (n = 50; Normality test: p < 0.001; Constant variance test: p < 0.001 ; r = 0.901, p < 0.001), BNST vs. mPFC (n = 50; Normality test: p < 0.001; Constant variance test: p < 0.001 ; r = 0.969, p < 0.001), BNST vs. NAc (n = 50; Normality test: p < 0.001; Constant variance test: p < 0.001; r = 0.941, p < 0.001), vHPC vs. mPFC (n = 50; Normality test: p < 0.001; Constant variance test: p < 0.001; r = 0.923, p < 0.001), vHPC vs. NAc (n = 50; Normality test: p < 0.001; Constant variance test: p < 0.001; r = 0.930, p < 0.001) and mPFC vs. NAc (n = 50; Normality test: p < 0.001; Constant variance test: p < 0.001; r = 0.968, p < 0.001).

Table S1

Input weights of brain-wide afferents to Vglut2^{+} BNST-, vHPC-, mPFC- and NAc-projecting BLA neurons

Abbreviation	Brain substructure	BNST	vHPC	mPFC	NAc	Classification
MO	Motor Cortex	0.42±0.13	0.14±0.07	0.11±0.07	0.32±0.22	Sensory
SS	Somatosensory cortex	0.25±0.08	0.18±0.09	0.21±0.10	0.67±0.33	Sensory
GU	Gustatory cortex	0.48±0.15	0.13±0.05	0.19±0.04	0.74±0.36	Sensory
VISC	Visceral area	0.17±0.06	0.05±0.02	0.22±0.10	0.38±0.17	Sensory
AUD	Auditory cortex	1.90±0.93	2.52±0.76	2.20±1.02	3.51±2.20	Sensory
VIS	Visual cortex	0.15±0.06	0.17±0.09	0.11±0.08	0.51±0.26	Sensory
OB	Olfactory bulb	0.05±0.03	0.01±0.01	0.04±0.03	0.04±0.04	Sensory
AON	Anterior olfactory nucleus	0.12±0.04	0.03±0.02	0.05±0.02	0.12±0.08	Sensory
LOT	Nucleus of the lateral olfactory tract	0.13±0.06	0.11±0.05	0.53±0.17	0.43±0.24	Sensory
TH	Thalamus	6.92±1.06	6.78±1.01	7.53±1.06	4.40±0.38	Sensory
ACC	Anterior cingulate cortex	0.13±0.06	0.07±0.03	0.29±0.06	0.09±0.05	Integrative
PL	Prelimbic cortex	0.44±0.15	0.22±0.07	1.06±0.23	0.15±0.05	Integrative
IL	Infralimbic cortex	0.29±0.07	0.33±0.13	0.61±0.22	0.07±0.04	Integrative
OFC	Orbitofrontal cortex	0.46±0.18	0.38±0.17	0.37±0.13	0.32±0.13	Integrative
AI	Agranular insular cortex	2.79±1.06	1.23±0.36	2.12±0.16	2.55±0.62	Integrative
RSC	Retrosplenial cortex	0.02±0.02	0.03±0.01	0.04±0.04	0.04±0.05	Integrative
PPC	Parieltal cortex	0.11±0.06	0.03±0.01	0.07±0.04	0.19±0.18	Integrative
TEa	Temporal association cortex	2.33±0.69	4.28±1.03	3.82±0.68	4.69±1.86	Integrative
PRC	Perirhinal cortex	1.13±0.40	0.54±0.16	2.52±0.48	0.73±0.42	Integrative
ECT	Ectorhinal area	2.02±0.49	3.08±0.97	3.13±0.19	2.75±0.47	Integrative
TT	Taenia tecta	0.18±0.06	0.09±0.07	0.07±0.02	0.12±0.07	Integrative
DP	Dorsal peduncular cortex	0.13±0.05	0.04±0.03	0.04±0.01	0.01±0.01	Integrative
PIR	Piriform cortex	8.97±0.52	11.12±1.95	9.65±1.30	12.03±2.22	Integrative
COA	Cortical amygdalar area	4.30±0.89	3.07±0.58	3.70±1.16	3.90±0.86	Integrative
PAA	Piriform-amygdalar area	3.78±0.70	3.04±0.47	2.79±0.91	3.74±0.91	Integrative
TR	Post piriform transition area	4.02±0.65	2.83±0.73	2.02±0.29	3.82±1.56	Integrative
CA1	Field CA1 of the hippocampus	13.91±1.27	16.91±2.84	16.95±1.55	10.27±1.47	Contextual
CA2	Field CA2 of the hippocampus	0.06±0.05	0.03±0.02	0.06±0.05	0.04±0.05	Contextual
CA3	Field CA3 of the hippocampus	0.59±0.24	1.20±0.42	1.40±0.71	0.25±0.21	Contextual
DG	Dentate gyrus	0.18±0.09	0.04±0.03	0.02±0.01	0.20±0.23	Contextual
SUB	Subiculum	13.52±1.60	17.81±2.22	12.39±2.14	14.11±3.94	Contextual
ENT	Entorhinal cortex	12.82±1.00	9.96±1.54	13.93±1.46	14.64±2.17	Contextual
HY	Hypothalamus	1.95±0.42	1.84±0.40	1.10±0.16	1.80±0.37	Neuromodulatory
VTA	Ventral tegmental area	0.08±0.03	0.06±0.03	0.10±0.01	0.07±0.03	Neuromodulatory
MS	Medial septal nucleus	0.21±0.05	0.17±0.05	0.09±0.04	0.03±0.03	Neuromodulatory
NDB	Diagonal band nucleus	0.67±0.18	0.36±0.07	0.39±0.12	0.70±0.15	Neuromodulatory

DR	Dorsal Raphe	0.19±0.05	0.24±0.09	0.32±0.12	0.18±0.07	Neuromodulatory
LC	Locus coeruleus	0.07±0.03	0.08±0.02	0.06±0.02	0.11±0.03	Neuromodulatory
STRd	Striatum dorsal region	0.65±0.25	0.65±0.19	0.76±0.15	1.19±0.39	Miscellaneous
STRv	Striatum ventral region	0.64±0.12	0.53±0.19	0.98±0.13	1.09±0.36	Miscellaneous
LSX	Lateral septal complex	0.07±0.05	0.13±0.07	0.06±0.04	0.33±0.16	Miscellaneous
sAMY	Striatum-like amygdalar nuclei	4.70±0.74	3.38±0.32	2.02±0.35	3.03±0.82	Miscellaneous
PALd	Pallidum, dorsal region	0.34±0.14	0.27±0.09	0.46±0.15	0.15±0.09	Miscellaneous
PALv	Pallidum, ventral region	2.94±0.63	2.43±0.28	2.78±0.58	1.66±0.21	Miscellaneous
PALm	Pallidum, medial region (ex. MS & NDB)	0.02±0.02	0.01±0.01	0.00±0.00	0.02±0.02	Miscellaneous
PALc	Pallidum, caudal region	0.73±0.29	0.31±0.11	0.38±0.10	0.40±0.23	Miscellaneous
MBsen	Midbrain, sensory-related	0.00±0.00	0.09±0.05	0.01±0.01	0.19±0.23	Miscellaneous
MBmot	Midbrain, motor-related (ex. VTA)	0.72±0.30	0.46±0.09	0.27±0.07	0.46±0.35	Miscellaneous
MBsta	Midbrain, behavioral state-related (ex. DR)	0.01±0.01	0.02±0.01	0.01±0.01	0.03±0.00	Miscellaneous
SP	Cortical subplate	3.23±0.59	2.52±0.45	1.97±0.62	2.72±0.57	Miscellaneous

Table S2

Relative input weights of brain-wide afferents to Vglut2$^+$ BLA output neurons

BLA neurons	Sensory	Integrative	*U* and *p* values
BNST-projecting	1.058±0.278, *n*=70	1.944±0.252, *n*=112	*U*=4924.0, *p*=0.004
vHPC-projecting	1.012±0.274, *n*=70	1.899±0.305, *n*=112	*U*=4906.5, *p*=0.004
mPFC-projecting	1.119±0.320, *n*=60	2.019±0.275, *n*=96	*U*=3860.5, *p*<0.001
NAc-projecting	1.113±0.270, *n*=50	2.200±0.387, *n*=80	*U*=2181.5, *p*=0.385

BLA neurons	Sensory	Contextual	*U* and *p* values
BNST-projecting	1.058±0.278, *n*=70	6.845±1.088, *n*=42	*U*=900.5, *p*<0.001
vHPC-projecting	1.012±0.274, *n*=70	7.660±1.345, *n*=42	*U*=853.0, *p*<0.001
mPFC-projecting	1.119±0.320, *n*=60	7.457±1.293, *n*=36	*U*=696.0, *p*=0.003
NAc-projecting	1.113±0.270, *n*=50	6.585±1.390, *n*=30	*U*=582.5, *p*=0.096

BLA neurons	Sensory	Neuromodulatory	*U* and *p* values
BNST-projecting	1.058±0.278, *n*=70	0.529±0.127, *n*=42	*U*=1496.0, *p*=0.878
vHPC-projecting	1.012±0.274, *n*=70	0.457±0.117, *n*=42	*U*=1218.0, *p*=0.128
mPFC-projecting	1.119±0.320, *n*=60	0.345±0.071, *n*=36	*U*=1070.9, *p*=0.942
NAc-projecting	1.113±0.270, *n*=50	0.483±0.137, *n*=30	*U*=883.5, *p*=0.185

BLA neurons	Sensory	Miscellaneous	*U* and *p* values
BNST-projecting	1.058±0.278, *n*=70	1.172±0.190, *n*=84	*U*=3174.5, *p*=0.393
vHPC-projecting	1.012±0.274, *n*=70	0.899±0.134, *n*=84	*U*=3555.5, *p*=0.025
mPFC-projecting	1.119±0.320, *n*=60	0.808±0.130, *n*=72	*U*=2381.5, *p*=0.308
NAc-projecting	1.113±0.270, *n*=50	0.938±0.157, *n*=60	*U*=1432.5, *p*=0.686

BLA neurons	Integrative	Contextual	*U* and *p* values
BNST-projecting	1.944±0.252, *n*=112	6.845±1.088, *n*=42	*U*=1731.5, *p*=0.012
vHPC-projecting	1.899±0.305, *n*=112	7.660±1.345, *n*=42	*U*=1673.0, *p*=0.006
mPFC-projecting	2.019±0.275, *n*=96	7.457±1.293, *n*=36	*U*=1354.5, *p*=0.056
NAc-projecting	2.200±0.387, *n*=80	6.585±1.390, *n*=30	*U*=956.5, *p*=0.101

BLA neurons	Integrative	Neuromodulatory	*U* and *p* values
BNST-projecting	1.944±0.252, *n*=112	0.529±0.127, *n*=42	*U*=3068.0, *p*=0.004
vHPC-projecting	1.899±0.305, *n*=112	0.457±0.117, *n*=42	*U*=2798.0, *p*=0.070
mPFC-projecting	2.019±0.275, *n*=96	0.345±0.071, *n*=36	*U*=2461.0, *p*<0.001
NAc-projecting	2.200±0.387, *n*=80	0.483±0.137, *n*=30	*U*=1506.0, *p*=0.040

BLA neurons	Integrative	Miscellaneous	*U* and *p* values
BNST-projecting	1.944±0.252, *n*=112	1.172±0.190, *n*=84	*U*=5501.5, *p*=0.042
vHPC-projecting	1.899±0.305, *n*=112	0.899±0.134, *n*=84	*U*=5148.5, *p*=0.256
mPFC-projecting	2.019±0.275, *n*=96	0.808±0.130, *n*=72	*U*=4497.5, *p*<0.001
NAc-projecting	2.200±0.387, *n*=80	0.938±0.157, *n*=60	*U*=2795.5, *p*=0.094

BLA neurons	Contextual	Neuromodulatory	*U* and *p* values
BNST-projecting	6.845±1.088, *n*=42	0.529±0.127, *n*=42	*U*=538.5, *p*=0.002
vHPC-projecting	7.660±1.345, *n*=42	0.457±0.117, *n*=42	*U*=564.5, *p*=0.004
mPFC-projecting	7.457±1.293, *n*=36	0.345±0.071, *n*=36	*U*=424.0, *p*=0.012
NAc-projecting	6.585±1.390, *n*=30	0.483±0.137, *n*=30	*U*=317.0, *p*=0.049

BLA neurons	Contextual	Miscellaneous	*U* and *p* values
BNST-projecting	6.845±1.088, *n*=42	1.172±0.190, *n*=84	*U*=1120.0, *p*<0.001
vHPC-projecting	7.660±1.345, *n*=42	0.899±0.134, *n*=84	*U*=1176.0, *p*=0.002
mPFC-projecting	7.457±1.293, *n*=36	0.808±0.130, *n*=72	*U*=840.5, *p*=0.003
NAc-projecting	6.585±1.390, *n*=30	0.938±0.157, *n*=60	*U*=642.0, *p*=0.026

BLA neurons	Neuromodulatory	Miscellaneous	*U* and *p* values
BNST-projecting	0.529±0.127, *n*=42	1.172±0.190, *n*=84	*U*=1983.5, *p*=0.253
vHPC-projecting	0.457±0.117, *n*=42	0.899±0.134, *n*=84	*U*=2033.5, *p*=0.162
mPFC-projecting	0.345±0.071, *n*=36	0.808±0.130, *n*=72	*U*=1463.5, *p*=0.273
NAc-projecting	0.483±0.137, *n*=30	0.938±0.157, *n*=60	*U*=1014.5, *p*=0.325

Table S3

Input weights of brain-wide afferents to Thy1$^+$ BNST-, vHPC-, mPFC- and NAc-projecting BLA neurons

Abbreviation	Brain substructure	BNST	vHPC	mPFC	NAc	Classification
MO	Motor Cortex	0.53±0.28	0.19±0.08	1.01±0.44	0.42±0.14	Sensory
SS	Somatosensory cortex	0.69±0.30	0.62±0.40	1.28±0.53	0.19±0.06	Sensory
GU	Gustatory cortex	0.39±0.22	0.09±0.05	0.22±0.12	0.16±0.12	Sensory
VISC	Visceral area	0.18±0.11	0.07±0.02	0.09±0.05	0.09±0.05	Sensory
AUD	Auditory cortex	0.67±0.50	2.04±0.67	1.98±0.55	0.61±0.11	Sensory
VIS	Visual cortex	0.28±0.25	0.23±0.09	0.78±0.29	0.16±0.10	Sensory
OB	Olfactory bulb	0.04±0.02	0.01±0.01	0.03±0.02	0.01±0.01	Sensory
AON	Anterior olfactory nucleus	0.07±0.02	0.26±0.20	0.14±0.04	0.16±0.04	Sensory
LOT	Nucleus of the lateral olfactory tract	0.25±0.06	0.28±0.06	0.16±0.06	0.17±0.06	Sensory
TH	Thalamus	4.30±0.86	5.06±0.84	5.98±1.21	6.46±0.36	Sensory
ACC	Anterior cingulate cortex	0.05±0.03	0.05±0.02	0.33±0.16	0.43±0.28	Integrative
PL	Prelimbic cortex	0.15±0.07	0.33±0.15	1.18±0.32	0.57±0.15	Integrative
IL	Infralimbic cortex	0.15±0.07	0.33±0.17	0.22±0.09	0.74±0.15	Integrative
OFC	Orbitofrontal cortex	0.28±0.11	0.42±0.12	1.41±0.22	0.65±0.18	Integrative
AI	Agranular insular cortex	1.96±0.88	0.87±0.28	1.47±0.40	1.40±0.70	Integrative
RSC	Retrosplenial cortex	0.02±0.01	0.02±0.01	0.01±0.01	0.03±0.01	Integrative
PPC	Parieltal cortex	0.14±0.08	0.09±0.04	0.23±0.09	0.07±0.03	Integrative
TEa	Temporal association cortex	1.23±0.25	4.35±0.81	3.30±0.25	1.95±0.42	Integrative
PRC	Perirhinal cortex	0.68±0.18	2.22±0.76	1.66±0.18	1.44±0.35	Integrative
ECT	Ectorhinal area	1.20±0.17	4.61±1.60	2.73±0.33	1.81±0.36	Integrative
TT	Taenia tecta	0.12±0.06	0.26±0.09	0.23±0.05	0.38±0.07	Integrative
DP	Dorsal peduncular cortex	0.04±0.02	0.08±0.03	0.05±0.03	0.16±0.07	Integrative
PIR	Piriform cortex	7.93±1.30	6.27±0.63	5.15±0.90	4.80±0.72	Integrative
COA	Cortical amygdalar area	6.11±1.17	2.78±0.73	4.09±0.74	4.50±0.87	Integrative
PAA	Piriform-amygdalar area	1.69±0.42	1.09±0.35	1.78±0.52	2.54±0.58	Integrative
TR	Post piriform transition area	1.71±0.25	2.59±0.66	1.47±0.48	1.77±0.37	Integrative
CA1	Field CA1 of the hippocampus	12.24±4.82	12.65±2.64	10.13±2.13	9.73±1.57	Contextual
CA2	Field CA2 of the hippocampus	1.81±0.57	1.36±0.73	0.99±0.32	0.67±0.39	Contextual
CA3	Field CA3 of the hippocampus	5.27±1.22	6.96±2.21	4.19±1.29	3.78±1.17	Contextual
DG	Dentate gyrus	0.04±0.02	0.05±0.02	0.40±0.26	0.14±0.13	Contextual
SUB	Subiculum	21.24±3.89	13.82±0.79	20.02±2.96	18.47±1.57	Contextual
ENT	Entorhinal cortex	6.12±0.85	10.30±1.23	6.80±0.99	11.23±2.86	Contextual
HY	Hypothalamus	3.37±0.70	3.31±0.85	3.18±0.65	3.67±0.83	Neuromodulatory
VTA	Ventral tegmental area	0.05±0.02	0.10±0.02	0.07±0.02	0.32±0.21	Neuromodulatory
MS	Medial septal nucleus	0.45±0.19	0.42±0.14	0.10±0.03	0.34±0.11	Neuromodulatory

NDB	Diagonal band nucleus	0.59±0.10	0.40±0.09	0.61±0.11	0.62±0.16	Neuromodulatory
DR	Dorsal Raphe	0.24±0.08	0.14±0.03	0.17±0.04	0.13±0.05	Neuromodulatory
LC	Locus coeruleus	0.05±0.02	0.14±0.10	0.03±0.01	0.02±0.01	Neuromodulatory
STRd	Striatum dorsal region	1.53±0.71	2.52±0.45	2.00±0.73	1.99±0.55	Miscellaneous
STRv	Striatum ventral region	0.57±0.10	1.14±0.29	0.90±0.21	2.35±0.56	Miscellaneous
LSX	Lateral septal complex	0.25±0.06	0.37±0.12	0.11±0.03	0.53±0.15	Miscellaneous
sAMY	Striatum-like amygdalar nuclei	7.73±1.16	4.33±0.44	4.66±0.71	4.40±1.11	Miscellaneous
PALd	Pallidum, dorsal region	0.14±0.02	0.43±0.11	0.43±0.12	0.40±1.11	Miscellaneous
PALv	Pallidum, ventral region	2.03±0.60	2.01±0.25	2.61±0.60	2.46±0.54	Miscellaneous
PALm	Pallidum, medial region (ex. MS & NDB)	0.77±0.30	0.61±0.19	0.63±0.25	0.76±0.25	Miscellaneous
PALc	Pallidum, caudal region	1.17±0.29	0.43±0.10	0.64±0.10	1.58±0.43	Miscellaneous
MBsen	Midbrain, sensory-related	0.03±0.01	0.31±0.14	0.03±0.02	0.07±0.02	Miscellaneous
MBmot	Midbrain, motor-related (ex. VTA)	0.71±0.14	1.02±0.31	1.61±0.86	1.44±0.29	Miscellaneous
MBsta	Midbrain, behavioral state-related (ex. DR)	0.17±0.09	0.02±0.01	0.02±0.01	0.01±0.01	Miscellaneous
SP	Cortical subplate	2.59±0.35	1.99±0.52	2.67±0.46	3.21±0.53	Miscellaneous

Table S4

Relative input weights of brain-wide afferents to Thy1⁺ BLA output neurons

BLA neurons	Sensory	Integrative	*U* and *p* values
BNST-projecting	0.738±0.180, *n*=70	1.465±0.242, *n*=112	*U*=4684.5, *p*=0.027
vHPC-projecting	0.884±0.211, *n*=70	1.647±0.226, *n*=112	*U*=4912.0, *p*=0.004
mPFC-projecting	1.166±0.251, *n*=70	1.582±0.168, *n*=112	*U*=4907.0, *p*=0.004
NAc-projecting	0.843±0.248, *n*=60	1.453±0.174, *n*=96	*U*=4153.5, *p*<0.001

BLA neurons	Sensory	Contextual	*U* and *p* values
BNST-projecting	0.738±0.180, *n*=70	7.786±1.497, *n*=42	*U*=581.0, *p*<0.001
vHPC-projecting	0.884±0.211, *n*=70	7.522±1.018, *n*=42	*U*=656.0, *p*<0.001
mPFC-projecting	1.166±0.251, *n*=70	7.090±1.215, *n*=42	*U*=676.5, *p*<0.001
NAc-projecting	0.843±0.248, *n*=60	7.337±1.248, *n*=36	*U*=510.5, *p*<0.001

BLA neurons	Sensory	Neuromodulatory	*U* and *p* values
BNST-projecting	0.738±0.180, *n*=70	0.792±0.216, *n*=42	*U*=1233.5, *p*=0.155
vHPC-projecting	0.884±0.211, *n*=70	0.750±0.226, *n*=42	*U*=1321.5, *p*=0.374
mPFC-projecting	1.166±0.251, *n*=70	0.694±0.204, *n*=42	*U*=1615.5, *p*=0.383
NAc-projecting	0.843±0.248, *n*=60	0.850±0.255, *n*=36	*U*=959.5, *p*=0.363

BLA neurons	Sensory	Miscellaneous	*U* and *p* values
BNST-projecting	0.738±0.180, *n*=70	1.476±0.255, *n*=84	*U*=4037.5, *p*<0.001
vHPC-projecting	0.884±0.211, *n*=70	1.265±0.152, *n*=84	*U*=4054.0, *p*<0.001
mPFC-projecting	1.166±0.251, *n*=70	1.360±0.192, *n*=84	*U*=3457.5, *p*=0.060
NAc-projecting	0.843±0.248, *n*=60	1.600±0.200, *n*=72	*U*=3154.0, *p*<0.001

BLA neurons	Integrative	Contextual	*U* and *p* values
BNST-projecting	1.465±0.242, *n*=112	7.786±1.497, *n*=42	*U*=1157.0, *p*<0.001
vHPC-projecting	1.647±0.226, *n*=112	7.522±1.018, *n*=42	*U*=1279.5, *p*<0.001
mPFC-projecting	1.582±0.168, *n*=112	7.090±1.215, *n*=42	*U*=1325.0, *p*<0.001
NAc-projecting	1.453±0.174, *n*=96	7.337±1.248, *n*=36	*U*=1045.5, *p*<0.001

BLA neurons	Integrative	Neuromodulatory	*U* and *p* values
BNST-projecting	1.465±0.242, *n*=112	0.792±0.216, *n*=42	*U*=2537.0, *p*=0.453
vHPC-projecting	1.647±0.226, *n*=112	0.750±0.226, *n*=42	*U*=2833.5, *p*=0.051
mPFC-projecting	1.582±0.168, *n*=112	0.694±0.204, *n*=42	*U*=3261.0, *p*<0.001
NAc-projecting	1.453±0.174, *n*=96	0.850±0.255, *n*=36	*U*=2316.5, *p*=0.003

BLA neurons	Integrative	Miscellaneous	*U* and *p* values
BNST-projecting	1.465±0.242, *n*=112	1.476±0.255, *n*=84	*U*=4089.0, *p*=0.117
vHPC-projecting	1.647±0.226, *n*=112	1.265±0.152, *n*=84	*U*=4333.0, *p*=0.346
mPFC-projecting	1.582±0.168, *n*=112	1.360±0.192, *n*=84	*U*=5166.5, *p*=0.240
NAc-projecting	1.453±0.174, *n*=96	1.600±0.200, *n*=72	*U*=3235.0, *p*=0.480

BLA neurons	Contextual	Neuromodulatory	*U* and *p* values
BNST-projecting	7.786±1.497, *n*=42	0.792±0.216, *n*=42	*U*=387.0, *p*<0.001
vHPC-projecting	7.522±1.018, *n*=42	0.750±0.226, *n*=42	*U*=408.0, *p*<0.001
mPFC-projecting	7.090±1.215, *n*=42	0.694±0.204, *n*=42	*U*=352.0, *p*<0.001
NAc-projecting	7.337±1.248, *n*=36	0.850±0.255, *n*=36	*U*=323.5, *p*<0.001

BLA neurons	Contextual	Miscellaneous	*U* and *p* values
BNST-projecting	7.786±1.497, *n*=42	1.476±0.255, *n*=84	*U*=962.0, *p*<0.001
vHPC-projecting	7.522±1.018, *n*=42	1.265±0.152, *n*=84	*U*=955.5, *p*<0.001
mPFC-projecting	7.090±1.215, *n*=42	1.360±0.192, *n*=84	*U*=352.0, *p*<0.001
NAc-projecting	7.337±1.248, *n*=36	1.600±0.200, *n*=72	*U*=793.0, *p*=0.001

BLA neurons	Neuromodulatory	Miscellaneous	*U* and *p* values
BNST-projecting	0.792±0.216, *n*=42	1.476±0.255, *n*=84	*U*=2233.0, *p*=0.015
vHPC-projecting	0.750±0.226, *n*=42	1.265±0.152, *n*=84	*U*=2393.5, *p*=0.001
mPFC-projecting	0.694±0.204, *n*=42	1.360±0.192, *n*=84	*U*=2307.5, *p*=0.005
NAc-projecting	0.850±0.255, *n*=36	1.600±0.200, *n*=72	*U*=1765.0, *p*=0.002
